# Homocysteine Promotes the Pathogenesis of Atherosclerosis through the Circ‐PIAS1‐5/miR‐219a‐2‐3p/TEAD1 Axis

**DOI:** 10.1002/advs.202415563

**Published:** 2025-03-16

**Authors:** Shengchao Ma, Fei Ma, Ning Ding, Lin Xie, Anning Yang, Jiangyong Shen, Yun jiao, Kai Wu, YueE Chai, Zhigang Bai, Jiantuan Xiong, Nan Li, Huiping Zhang, Yideng Jiang

**Affiliations:** ^1^ NHC Key Laboratory of Metabolic Cardiovascular Diseases Research Ningxia Medical University Yinchuan 750004 China; ^2^ Ningxia Key Laboratory of Vascular Injury and Repair Research Ningxia Medical University Yinchuan 750004 China; ^3^ School of Basic Medical Sciences Ningxia Medical University Yinchuan 750004 China; ^4^ General Hospital of Ningxia Medical University Yinchuan 750004 China; ^5^ Clinical Medical School Ningxia Medical University Yinchuan 750004 China; ^6^ People's Hospital of Ningxia Hui Autonomous Region Yinchuan 750004 China; ^7^ Maternal and Child Health of Hunan Province Changsha 410008 China

**Keywords:** atherosclerosis, circ‐PIAS1‐5, homocysteine, miR‐219a‐2‐3p, YTHDC1

## Abstract

Previous studies have established a possible link between hyperhomocysteinemia (HHcy) and dyslipidemia. Circular RNAs (circRNAs) play important regulatory roles in the development of atherosclerosis. However, the biological functions and potential molecular mechanisms of circRNAs in HHcy‐induced lipid accumulation leading to atherosclerosis are still unclear. In this study, it is determined that homocysteine (Hcy) downregulates the expression of circ‐PIAS1‐5 by global circRNA expression profiling and that circ‐PIAS1‐5 inhibits Hcy‐mediated lipid accumulation in foam cells and the pathogenesis of atherosclerosis by acting as a sponge for miR‐219a‐2‐3p. Circ‐PIAS1‐5 is identified as a potential diagnostic biomarker of HHcy‐associated atherosclerosis in male “apolipoprotein E knockout (ApoE^−/−^)” mice. Mechanistically, circ‐PIAS1‐5 activates the adenosine 5‘‐monophosphate (AMP)‐activated protein kinase pathway by regulating TEAD1 through miR‐219a‐2‐3p, and Hcy mediates the m^6^A modification and nuclear export of circ‐PIAS1‐5 via YTHDC1 to increase lipid accumulation in foam cells and accelerate the pathogenesis of atherosclerosis. Taken together, these results highlight the role of circ‐PIAS1‐5 in the Hcy‐mediated pathogenesis of atherosclerosis and suggest its potential application as a prognostic biomarker of atherosclerosis induced by HHcy.

## Graphical abstract

A model is schematically represented for the major molecular mechanisms by which homocysteine (Hcy) accelerates the nuclear export of circ‐PIAS1‐5, which regulates atherosclerosis by acting as a competing endogenous RNA for miR‐219a‐2‐3p. Hcy enrichment of circ‐PIAS1‐5 leads to YTHDC1 binding to circ‐PIAS1‐5 and promotes its intracellular localization from the nucleus to the cytoplasm, which contributes to circ‐PIAS1‐5 acting as a sponge of miR‐219a‐5p to promote lipid accumulation in foam cells in Hcy‐induced atherosclerosis through regulating TEAD1.

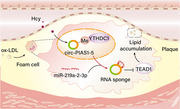

## 1 Introduction

Atherosclerosis is a common and important pathological factor of cardiovascular disease (CVD).^[^ ^1^ ^]^ It is characterized by lipid accumulation on the arterial wall followed by intraplaque hemorrhage, plaque rupture, and neoplasia, causing severe ischemia and necrosis of tissues and organ death.^[^ ^2^ ^]^ Modifications of plasma‐derived lipoproteins and their uptake by mainly macrophages, with the subsequent formation of lipid‐filled foam cells, initiate atherosclerotic lesion formation.^[^ ^3^, ^4^ ^]^ Homocysteine (Hcy), an intermediate metabolite of methionine, has been established as an independent risk factor for atherosclerosis.^[^ ^5^ ^]^ However, the mechanisms of Hcy‐induced lipid accumulation during atherosclerotic plaque formation have not yet been fully explored.

Circular RNAs (circRNAs), which have a circular configuration through typical 5′–3′ phosphodiester bonds, are recognized as a class of functional noncoding RNAs (ncRNAs).^[^ ^6^ ^]^ More than 20% of the genes expressed in the genome can produce circRNAs, which present notable abundance, stability, and diversity of expression profiles in various types of tissues and cells. For example, ≈85% of circRNAs are highly tissue specific, which, in theory, suggests that they may serve as disease markers or therapeutic targets.^[^ ^7^, ^8^ ^]^ Mechanistically, circRNAs can be used as microRNA (miRNA)‐ or RNA‐binding protein decoys to regulate gene expression or protein translation and thus play pivotal roles in various physiological activities and diseases.^[^ ^9^ ^]^ More importantly, circRNAs fine‐tune post‐transcription via their interactions with miRNAs or with single or multiple RNA‐binding proteins.^[^ ^10^ ^]^ Many attempts to comprehensively identify atherosclerotic plaque constituents with different invasive and noninvasive imaging technologies have been made and provide great help for atherosclerotic plaque diagnosis and treatment.^[^ ^11^ ^]^ The use of these technologies, however, has gradually led to certain issues, such as high costs, low patient acceptance, and the inability to achieve personalized diagnosis. Therefore, the search for more convenient and personalized diagnostic criteria is becoming increasingly important. More recently, a series of circulating circRNAs have been identified to act as both essential regulatory molecules and biomarkers for the progression of metabolism‐related disorders, including type 2 diabetes mellitus (T2DM or T2D) and CVD.^[^ ^12^ ^]^ Using high‐throughput RNA sequencing technology, recent discoveries have shown that many circRNAs are extensively expressed and associated with fatty acid metabolism or lipid deposition.^[^ ^13^ ^]^ However, the function of circRNAs in Hcy‐induced lipid accumulation during atherosclerotic plaque formation remains unclear. It is well known that circRNAs have high stability and can be used as new biomarkers for disease diagnosis or prognosis. Therefore, exploring the potential mechanism of atherosclerosis induced by Hcy, which may lead to the development of new biomarkers and treatment strategies, is highly important.

N6‐methyladenosine (m^6^A), the first identified mammalian messenger RNA (mRNA) modification and the most abundant modification known in eukaryotic mRNAs and ncRNAs, regulates RNA transcription, processing, splicing, degradation, and translation, which have cell‐ and tissue‐wide effects.^[^ ^14^ ^]^ The dynamic m^6^A modification of mRNAs within the nucleus has emerged as a primary regulator of RNA structure. As the main m^6^A reader, YT521‐B homology (YTH) domain‐containing proteins (YTHDF1‐3 and YTHDC1‐2) bind m^6^A‐modified RNAs to perform specific biological functions.^[^ ^15^, ^16^ ^]^ YTHDC1, a nuclear m^6^A reader, promotes the nuclear export of m^6^A‐modified cellular RNAs, accelerates the decay of certain transcripts, and regulates mRNA splicing by recruiting certain splicing factors.^[^ ^17^ ^]^ A recent study revealed that YTHDC1 can selectively identify m^6^A‐modified RNA and then be delivered to the nuclear export factor 1 (NXF1)‐SR protein family (SRSF) complex to accelerate the nuclear export of its target RNA.^[^ ^18^ ^]^ Another study demonstrated that YTHDC1 recognizes m^6^A‐modified X‐inactive specific transcript (XIST) to promote XIST‐mediated X chromosome silencing.^[^ ^19^ ^]^ However, the specific role and corresponding mechanism of YTHDC1 in terms of m^6^A modifications have not been fully elucidated in Hcy‐induced atherosclerosis.

In this study, we identified and characterized a novel circ‐PIAS1‐5 (lipid‐related factor sequence) as an essential regulator of foam lipid accumulation in atherosclerosis in apolipoprotein E knockout (ApoE^−/−^) mice. More importantly, Hcy accelerates the nuclear export of circ‐PIAS1‐5, which regulates atherosclerosis, by acting as a competing endogenous RNA for miR‐219a‐2‐3p. Clinically, receiver operating characteristic (ROC) curve analysis revealed that the diagnostic value of circ‐PIAS1‐5 in atherosclerotic patients with high Hcy concentrations is highly effective. Thus, circ‐PIAS1‐5 might serve as a potential biomarker of atherosclerosis induced by Hcy, and it might be used in the early diagnosis, development, and prognosis of atherosclerosis.

## 2 Results

### 2.1 Identification of Circular RNAs by RNA‐seq Analyses in Foam Cells Treated with Hcy

**Figure 1:**
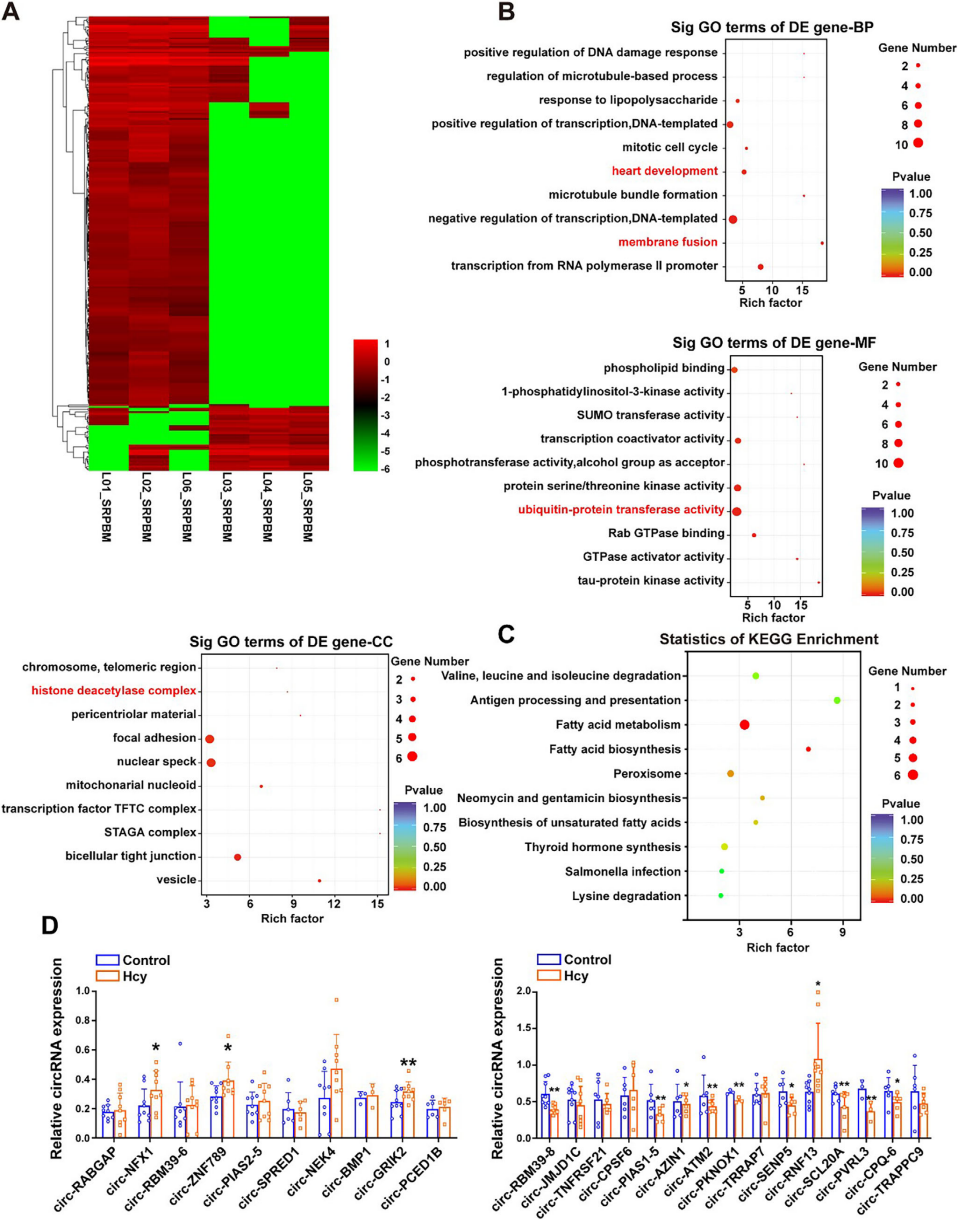
Differentially expressed circRNAs involved in the regulation of lipid accumulation in foam cells. A) Cluster heat map of all the differentially expressed circRNAs in foam cells treated with or without Hcy. The red and green strips represent upregulated and downregulated circRNAs, respectively. B) The top ten enriched biological process (BP), cellular component (CC), and molecular function (MF) terms were determined by Gene Ontology (GO) analysis. C) KEGG analysis of the top ten pathways related to lipid metabolism of differentially expressed circRNAs in foam cells treated with or without Hcy. D) qRT‐PCR analysis of 10 upregulated and 15 downregulated circRNAs in foam cells treated with Hcy. The data are presented as the mean ± standard deviation (SD). ^*^ *p* < 0.05, ^**^ *p* < 0.01, compared with the control group.

The transformation of macrophages into foam cells and subsequent lipid accumulation in foam cells play key roles in the pathogenesis of atherosclerosis, and Hcy is an independent risk factor for atherosclerosis. To further elucidate how Hcy contributes to the pathogenesis of atherosclerosis, we screened the differently expressed circRNAs in foam cells after Hcy treatment. The results revealed that Hcy upregulated the expression of 30 circRNAs and downregulated the expression of 58 circRNAs (**Figure** **1**A). Gene Ontology (GO) annotation analysis revealed that these circRNAs are involved in heart development, membrane fusion, the histone deacetylase complex, and ubiquitin‐protein transferase activity. Heart development and membrane fusion are reportedly associated with the progression of atherosclerosis. Knockout of histone deacetylase in endothelial cells can reduce plaque area and lipid accumulation in the pathogenesis of atherosclerosis, whereas ubiquitin‐protein transferase activity is recognized as a driving factor in the pathogenesis of atherosclerosis (Figure 1B). Furthermore, we specifically analyzed the pathways associated with lipid metabolism via Kyoto Encyclopedia of Genes and Genomes (KEGG), which revealed that the differentially expressed circRNAs involved in lipid metabolism, such as “fatty acid metabolism,” “steroid biosynthesis,” and the “adipocytokine signaling pathway” (Figure 1C). These findings suggest that the differentially expressed circRNAs might play a role in lipid accumulation in foam cells and the pathogenesis of atherosclerosis. From the overlap, we selected 15 downregulated and 10 upregulated lipid accumulation‐related circRNAs for experimental validation using real‐time quantitative polymerase chain reaction (qRT‐PCR). As shown in Figure 1D, circ‐PIAS1‐5 expression was significantly downregulated in foam cells, which was consistent with the sequencing data. Therefore, in this study, we focused on the role of circ‐PIAS1‐5 in Hcy‐induced atherosclerosis by affecting lipid accumulation in foam cells.

### 2.2 Validation of a Novel Circ‐PIAS1‐5 Involved in Lipid Accumulation in Foam Cells Treated with Hcy

**Figure 2:**
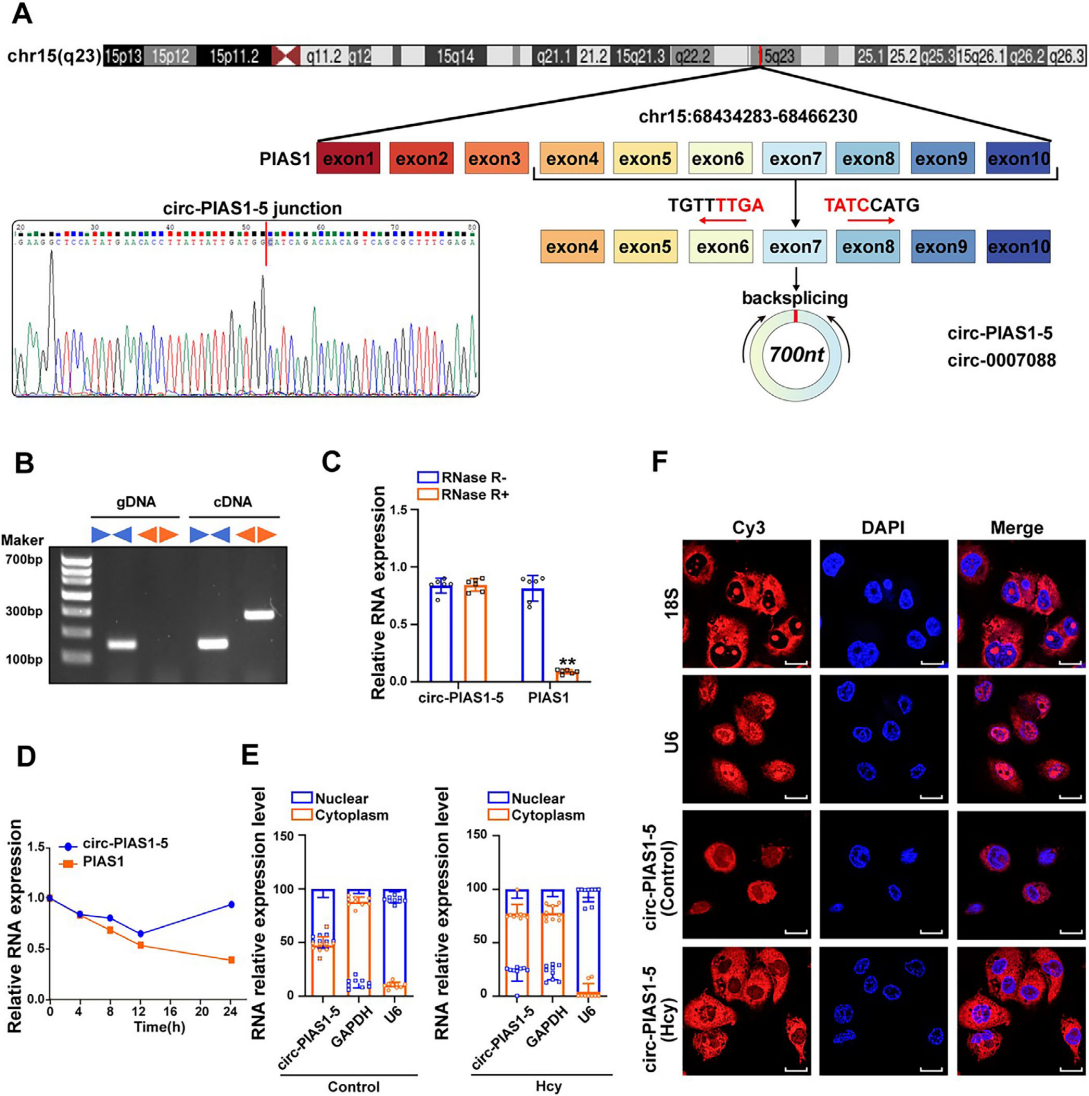
Identification and characterization of circ‐PIAS1‐5. A) Sanger sequencing was utilized to confirm that circ‐PIAS1‐5 is generated from exons 4 to 10 of PIAS1 by head‐to‐tail splicing. The red arrows represent divergent primers. B) Agarose gel electrophoresis showing that divergent primers amplified circ‐PIAS1‐5 in complementary DNA (cDNA) but not in genomic DNA (gDNA). C) The expression of circ‐PIAS1‐5 and PIAS1 was detected by qRT‐PCR in foam cells treated with or without RNase R. D) The relative RNA levels of circ‐PIAS1‐5 and linear PIAS1 mRNA in foam cells treated with actinomycin D at the indicated time points were determined v. E) Circ‐PIAS1‐5 levels in the cytoplasm and nucleus of foam cells were determined by qRT‐PCR analysis. glyceraldehyde‐3‐phosphate dehydrogenase (GAPDH) and U6 RNA were used as positive controls in the cytoplasm and nucleus, respectively. F) RNA fluorescence in situ hybridization for circ‐PIAS1‐5 was performed in foam cells treated with Hcy. Nuclei were stained with 4,6‐diamidino‐2‐phenylindole (DAPI) (blue), and circ‐PIAS1‐5 probes were labeled with Cy3 (red) (scale bar = 10 µm). The data are presented as the mean ± SD. **p < 0.01, compared with the RNase R^−^ group.

To clarify the origin of circ‐PIAS1‐5, we searched the UCSC Genome Browser (https://genome. ucsc.edu/index. html) and found that circ‐PIAS1‐5 was a circular transcript generated from the back splicing of exons 4–10 of the PIAS1 gene located on human chromosome 15 with a length of 700 nt, which was preliminarily confirmed by Sanger sequencing (**Figure** **2**A). Next, the covalent closed‐loop structure of endogenous circ‐PIAS1‐5 was verified by RT‐PCR with convergent and divergent primers. The results indicated that circ‐PIAS1‐5 was detected only in complementary DNA (cDNA), with no products detected in the extracted genomic DNA (gDNA), whereas the convergent primers amplified PIAS1 from both cDNA and gDNA (Figure 2B). After that, the stability of circ‐PIAS1‐5 in foam cells treated with RNase R or actinomycin D was analyzed. The qRT‐PCR results revealed that circ‐PIAS1‐5 had strong tolerance to RNase R digestion, whereas the linear form of PIAS1 was rapidly degraded (Figure 2C). A stability assay using actinomycin D treatment also demonstrated that circ‐PIAS1‐5 was substantially more stable than linear PIAS1 in foam cells (Figure 2D). Furthermore, separation of the cytoplasmic and nuclear fractions suggested that the subcellular localization of circ‐PIAS1‐5 was mainly in the cytoplasm (Figure 2E), which was further confirmed by RNA fluorescence in situ hybridization (RNA‐FISH, Figure 2F). Taken together, these initial results confirm the presence of circ‐PIAS1‐5 in foam cells and suggest a possible role for circ‐PIAS1‐5 in atherosclerosis induced by Hcy.

### 2.3 Circ‐PIAS1‐5 Inhibits Hcy‐Mediated Lipid Accumulation in Foam Cells in Atherosclerosis

**Figure 3:**
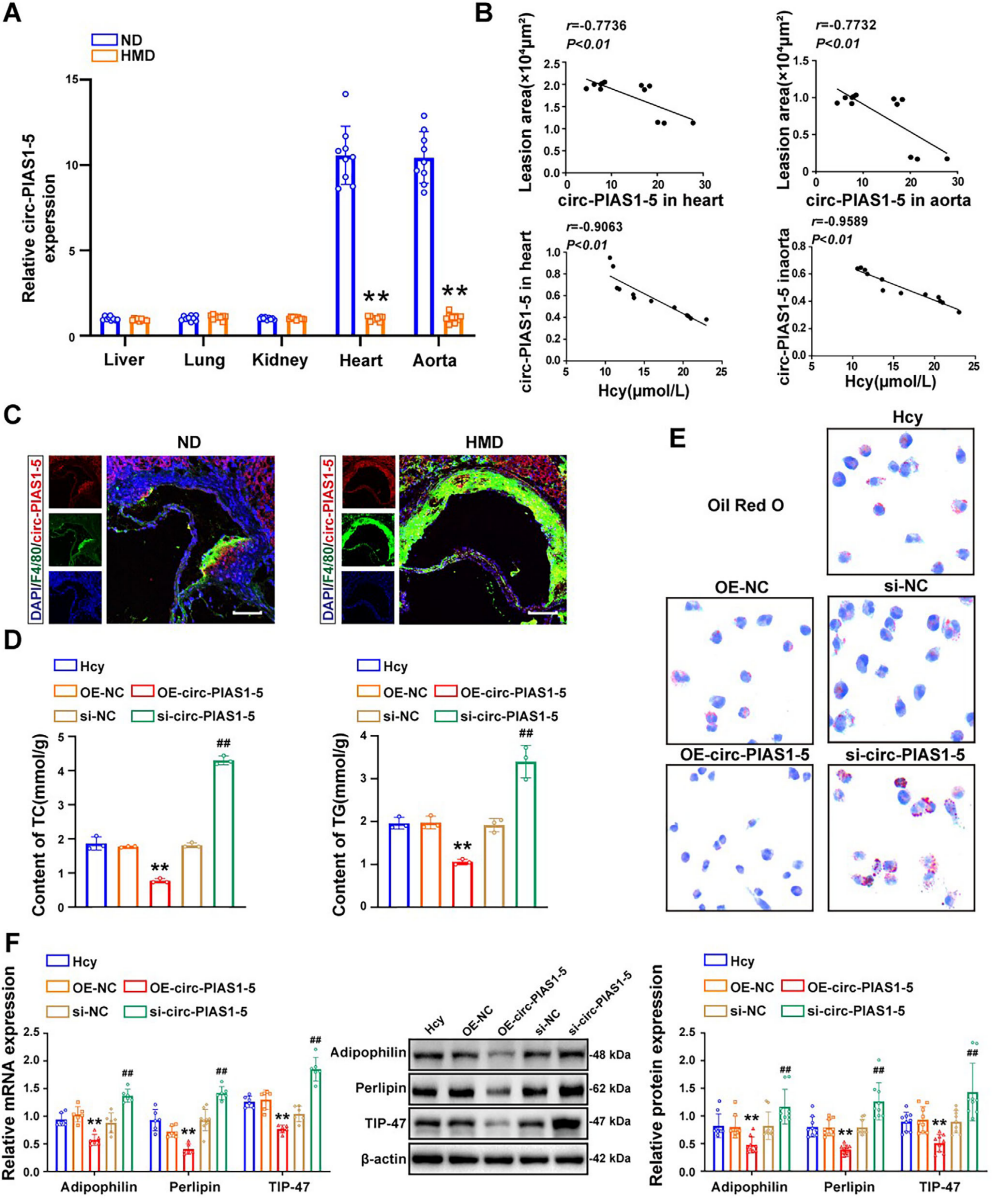
Circ‐PIAS1‐5 inhibits lipid accumulation in foam cells in atherosclerosis induced by Hcy. A) The expression of circ‐PIAS1‐5 in the heart, liver, lung, kidney, and aorta of ApoE^−/−^ mice was measured by qRT‐PCR. B) The correlations between atherosclerotic plaque areas and the expression of circ‐PIAS1‐5 in the heart and aorta of ApoE^−/−^ mice were calculated via Pearson's correlation analysis. C) RNA‐FISH and immunostaining revealed that circ‐PIAS1‐5 (red) co‐localization with F4/80 (green) in atherosclerotic plaques of ApoE^−/−^ mice fed a normal or high‐methionine diet (scale bar = 50 µm). D) TC and TG contents were detected in foam cells transfected with circ‐PIAS1‐5‐overexpressing vectors (OE‐circ‐PIAS1‐5) or siRNAs against circ‐PIAS1‐5 (si‐circ‐PIAS1‐5) in the presence of Hcy. E) Representative images of Oil Red O staining in foam cells transfected with OE‐circ‐PIAS1‐5 or si‐circ‐PIAS1‐5 in the presence of Hcy (scale bar = 50 µm). F) The expression of perilipin, adipophilin, and TIP47 in foam cells transfected with OE‐circ‐PIAS1‐5 or si‐circ‐PIAS1‐5 in the presence of Hcy was determined via western blotting and qRT‐PCR. The data are presented as the mean ± SD. ^**^ *p* < 0.01, compared with the ND group or the Hcy group. ^##^ *p* < 0.01, compared with the OE‐NC group or the si‐NC group.

To determine the expression pattern of circ‐PIAS1‐5, qRT‐PCR was used to detect the expression level of circ‐PIAS1‐5 in the liver, lung, kidney, heart, and aorta of ApoE^−/−^ mice. The results revealed that circ‐PIAS1‐5 was specifically decreased in the aorta and heart of ApoE^−/−^ mice fed a high‐methionine diet (**Figure** **3**A), and the serum Hcy concentration was markedly increased 3.71‐folds in mice fed a high‐methionine diet (*p* < 0.001) (Figure S1, Supporting Information), whereas a high‐methionine diet suppressed the body weight of ApoE^−/−^ mice (Figure S2, Supporting Information). Pearson's correlation analysis revealed that the expression of circ‐PIAS1‐5 in the aorta was negatively correlated with atherosclerotic plaque area, whereas the expression of circ‐PIAS1‐5 in the heart was also negatively correlated with the area of atherosclerotic plaque and have a negative correlation between serum Hcy level and heart and blood vessel circ‐PIAS1‐5 (Figure 3B). In addition, RNA‐FISH revealed increased co‐localization of circ‐PIAS1‐5 with F4/80 in the atherosclerotic plaques of ApoE^−/−^ mice fed a high‐methionine diet (Figure 3C). These results suggest that circ‐PIAS1‐5 may be involved in Hcy‐induced atherosclerosis in ApoE^−/−^ mice. To study the possible function of circ‐PIAS1‐5 in foam cells, we transfected circ‐PIAS1‐5‐overexpressing vectors (OE‐circ‐PIAS1‐5) and small interfering RNA (siRNAs) against circ‐PIAS1‐5 (si‐circ‐PIAS1‐5) into foam cells (Figure S3, Supporting Information). The triglyceride (TG) and total cholesterol (TC) contents were obviously decreased in foam cells transfected with OE‐circ‐PIAS1‐5, whereas increased TG and TC contents were detected in foam cells transfected with si‐circ‐PIAS1‐5 (Figure 3D). Consistently, Oil Red O staining revealed that lipid accumulation was markedly decreased in foam cells transfected with OE‐circ‐PIAS1‐5 but increased in foam cells transfected with si‐circ‐PIAS1‐5 (Figure 3E). Perilipin is the most abundant protein on the surface of adipocyte lipid droplets. TIP47 and adipophilin are not confined to the surface of lipid droplets, as expected, but are also located inside lipid droplet cores; both of these proteins are involved in intracellular lipid accumulation in cells.^[^ ^20^ ^]^ We also observed the expression of perilipin, adipophilin, and TIP47 in foam cells transfected with OE‐circ‐PIAS1‐5 or si‐circ‐PIAS1‐5 (Figure 3F), with and without Hcy, circ‐PIAS1‐5 participated in lipid accumulation in foam cells (Figure S3, Supporting Information). Taken together, these findings demonstrated that circ‐PIAS1‐5 inhibits Hcy‐mediated lipid accumulation in foam cells in atherosclerosis.

### 2.4 Upregulation of circ‐PIAS1‐5 Alleviates Atherosclerosis Induced by Hcy in ApoE^−/−^ Mice

**Figure 4:**
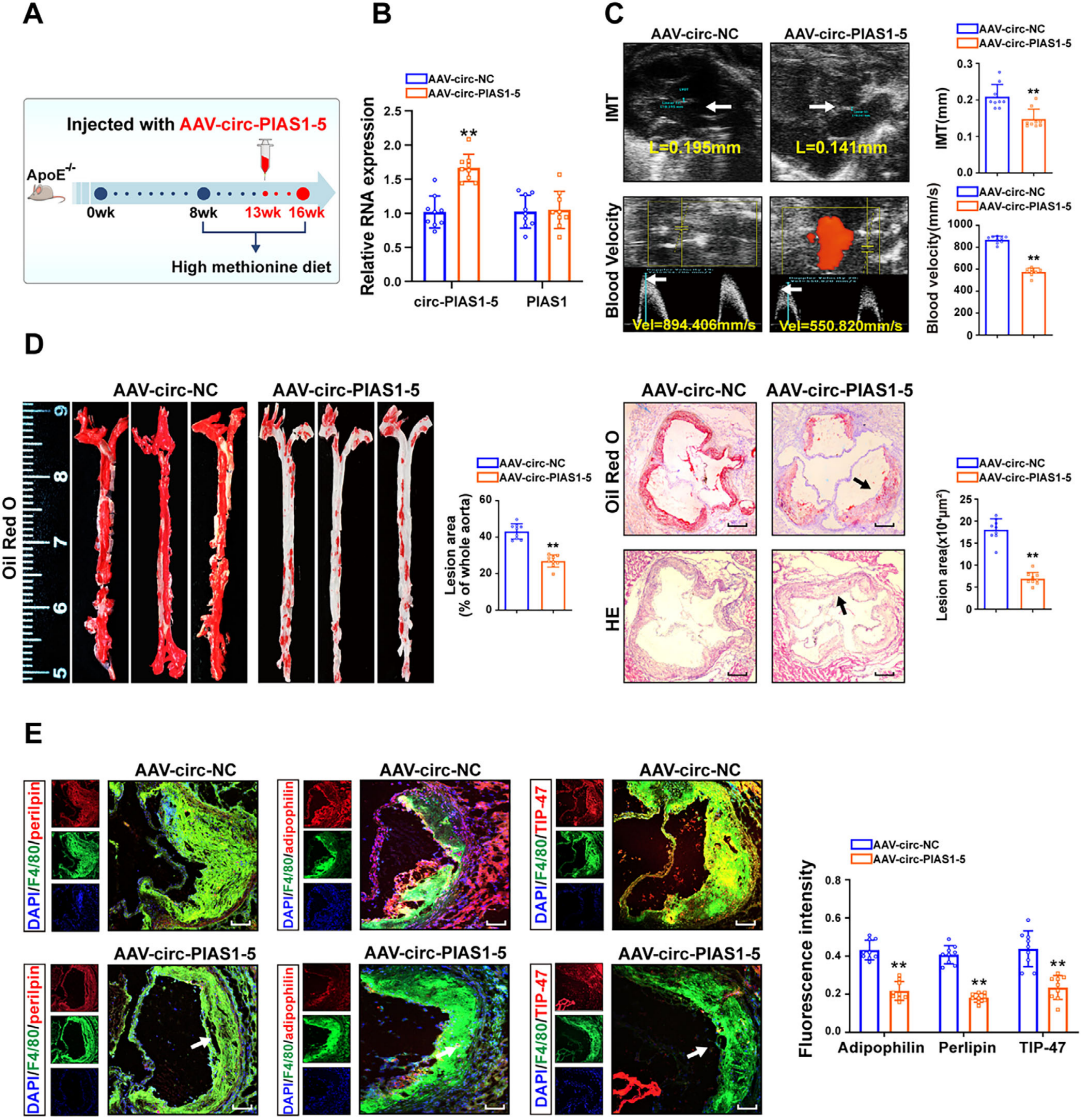
Circ‐PIAS1‐5 suppresses atherosclerosis induced by Hcy in ApoE^−/−^ mice. A) Male ApoE^−/−^ mice were intravenously injected with circ‐PIAS1‐5‐overexpressing adeno‐associated virus (AAV‐circ‐PIAS1‐5) or a mock vector with no circ‐PIAS1‐5 sequence (AAV‐circ‐NC) at 13 weeks and fed a high‐methionine diet for 3 weeks. B) The expression of circ‐PIAS1‐5 and PIAS1 in the aortas of ApoE^−/−^ mice was assessed by qRT‐qPCR. C) Representative images and quantification of the intima media thickness (IMT, yellow arrow) in the aortic root and the blood velocity (yellow arrow) in the ascending aorta of ApoE^−/−^ mice. D) Representative images of HE and Oil Red O staining of en face aortas and aortic sinus cross sections and quantitative analysis of the percentage of atherosclerotic plaque area in ApoE^−/−^ mice (scale bar = 500 µm). E) Representative immunofluorescence images and quantification of F4/80 (green) co‐localization with perilipin (red), adipophilin (red), and TIP47 (red) in the aortas of ApoE^−/−^ mice (scale bar = 50 µm). The data are presented as the mean ± SD. ^**^ *p* < 0.01, compared with the AAV‐circ‐NC group.

To further determine the physiological role of circ‐PIAS1‐5 in atherosclerosis induced by Hcy, circ‐PIAS1‐5‐overexpressing adeno‐associated virus (AAV‐circ‐PIAS1‐5) or a mock vector with no circ‐PIAS1‐5 sequence (AAV‐circ‐NC) was injected into ApoE^−/−^ mice fed a high‐methionine diet at 13 weeks (**Figure** **4**A). After 13 weeks, we performed qRT‐PCR to validate the expression of circ‐PIAS1‐5 and PIAS1‐5, and the results revealed that the expression of circ‐PIAS1‐5 was obviously increased in ApoE^−/−^ mice injected with AAV‐circ‐PIAS1‐5, whereas there was no change in PIAS1‐5 expression (Figure 4B). Notably, the ultrasound microscopy results revealed that the intima media thickness (IMT) of the aortic root and the blood velocity of the ascending aorta were significantly decreased in ApoE^−/−^ mice injected with AAV‐circ‐PIAS1‐5 (Figure 4C). Correspondingly, hematoxylin‐eosin staining (HE) and Oil Red O staining revealed that the atherosclerotic lesions of whole aortas and aortic roots were markedly decreased (Figure 4D). Immunofluorescence assays revealed that the co‐localization of perilipin, adipophilin, and TIP47 with F4/80 was significantly reduced in the atherosclerotic plaques of ApoE^−/−^ mice injected with AAV‐circ‐PIAS1‐5 (Figure 4E), which indicates that circ‐PIAS1‐5 attenuated Hcy‐induced atherosclerosis by suppressing lipid accumulation in ApoE^−/−^ mice.

### 2.5 Circ‐PIAS1‐5 Is a Potential Diagnostic Biomarker for Hyperhomocysteinemia‐Associated Atherosclerosis

**Figure 5:**
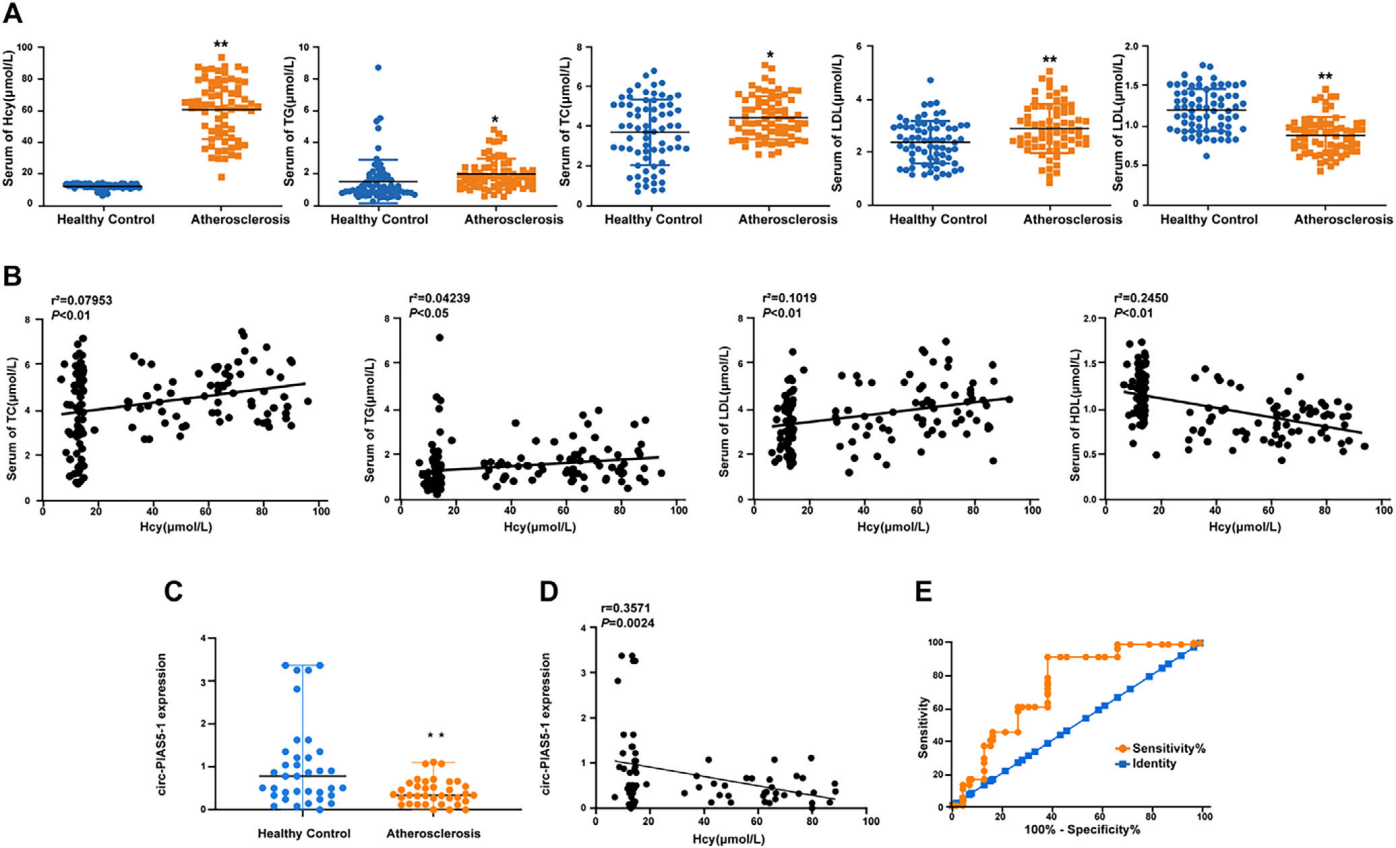
Diagnostic performance of circulating circ‐PIAS1‐5 for HHcy‐associated atherosclerosis. A) The serum levels of Hcy, TC, TG, LDL, and HDL in atherosclerotic patients with HHcy (atherosclerosis, *n* = 70) and healthy controls (*n* = 70) were detected by an automatic biochemical apparatus. B) Pearson's correlation analysis was used to analyze the linear correlation between the concentrations of Hcy and those of TC, TG, HDL, and LDL. C) The expression of circ‐PIAS1‐5 in monocytes in the peripheral blood of healthy controls and atherosclerotic patients with HHcy was determined by qRT‐PCR. D) The linear correlation between Hcy concentration and circ‐PIAS1‐5 expression in the blood of atherosclerotic patients was calculated by Pearson's correlation analysis. E) The cut‐off value, sensitivity, and specificity were established via receiver operating characteristic (ROC) curves to evaluate the diagnostic value of circ‐PIAS1‐5 for atherosclerotic patients with HHcy. The data are presented as the mean ± SD. ^*^ *p* < 0.05, ^**^ *p* < 0.01, compared with the healthy Control group.

To elucidate the diagnostic value and potential precision of circ‐PIAS1‐5 in the diagnosis of hyperhomocysteinemia (HHcy)‐associated atherosclerosis patients, we first enrolled a total of 140 individuals, including 70 healthy controls and 70 patients with atherosclerosis concomitant with HHcy (**Table** **1**). The statistical results of the clinical case data revealed that the serum levels of Hcy, TC, TG, and low‐density lipoprotein (LDL) were increased in patients with atherosclerosis concomitant with HHcy, whereas the high‐density lipoprotein (HDL) levels were decreased (**Figure** **5**A). Moreover, Pearson's correlation analysis revealed that the TC, TG, and LDL levels were positively correlated with the Hcy concentration (*r^2^* = 0.07953, *p* < 0.05; *r^2^* = 0.04239, *p* < 0.05; *r^2^* = 0.1019, *p* < 0.01, respectively), whereas the HDL level was negatively correlated with the Hcy concentration (*r^2^* = 0.2450, *p* < 0.01) (Figure 5B). Considering that there is a positive correlation between circ‐PIAS1‐5 expression in the plasma and aorta of ApoE^−/−^ mice fed a high‐methionine diet, we further elucidated whether circ‐PIAS1‐5 can be used as a novel diagnostic biomarker for HHcy‐associated atherosclerosis. The results revealed that circ‐PIAS1‐5 expression in the blood of patients with HHcy‐associated atherosclerosis was lower than that in the blood of healthy controls (Figure 5C); circ‐PIAS1‐5 exhibits a more intimate relationship with Hcy (**Table** **2**); and there was no significant difference in circ‐PIAS1‐5 expression between the atherosclerosis without HHcy group and the healthy group (Figure S4, Supporting Information). Pearson's correlation analysis revealed that circ‐PIAS1‐5 expression was negatively correlated with the Hcy concentration (*r^2^* = 0.1333, *p* < 0.0001) (Figure 5D). The area under curve (AUC) for atherosclerotic patients with HHcy was high, with 88.6% sensitivity and 75.6% specificity, and the AUC was 0.7336 (Figure 5E). Collectively, these results suggest that circ‐PIAS1‐5 might be a potential diagnostic marker for patients with atherosclerosis concomitant with HHcy.

**Table 1:** Demographic, clinical, and biochemical characteristics of study subjects (mean ± SD) (Hcy, homocysteine; TC, total cholesterol; TG, triglyceride; HDL, high‐density lipoprotein; LDL, low‐density lipoprotein; Glu, glucose; Crea, creatinine; ALB, albumin; AST, aspartate transaminase; ALT, alanine aminotransferase. Bold indicates statistical significance (*p* < 0.05)).

Features	Healthy controls (*n* = 70)	Atherosclerosis (*n* = 70)	*P*‐value
Male/Female (*n*/*n*)	32/38	34/36	
Age [years]	47.45 ± 17.66	51.15 ± 18.30	0.245
Hcy [µmol L^−1^]	12.93 ± 0.21	61.23 ± 2.21	<0.001
TG [mmol L^−1^]	1.61 ± 0.17	2.10 ± 0.12	0.0047
TC [mmol L^−1^]	3.92 ± 0.21	4.69 ± 0.14	0.0023
HDL [mmol L^−1^]	1.22 ± 0.03	0.90 ± 0.028	<0.0001
LDL [mmol L^−1^]	2.50 ± 0.10	2.77 ± 0.92	0.0006
Urea	4.61 ± 0.16	8.61 ± 2.14	0.0648
Crea	64.68 ± 8.65	63.96 ± 1.72	0.9423
ALB	44.10 ± 3.69	43.39 ± 4.90	0.175
AST	23.23 ± 12.05	70.87 ± 522.04	0.288
ALT	26.98 ± 19.85	69.33 ± 410.38	0.230
Blood glucose	5.34 ± 1.03	5.71 ± 1.51	0.243

**Table 2:** Correlation between the level of circPIAS1‐5 and clinicopathological characteristics of the participants (Hcy, homocysteine; TC, total Cholesterol; TG, triglyceride; HDL, high‐density lipoprotein; LDL, low‐density lipoprotein; Glu, glucose; Crea, creatinine; ALB, albumin; AST, aspartate transaminase; ALT, alanine aminotransferase. Bold indicates statistical significance (*p* < 0.05)).

Features	circ‐PIAS1‐5		*p*‐value
	Low (mean ± SD)	High (mean ± SD)	
Age [years]	47.45 ± 17.66	51.15 ± 18.30	0.245
Hcy	30.01 ± 26.51	53.02 ± 24.56	<0.001
TG	1.85 ± 1.63	1.85 ± 0.94	0.976
TC	4.55 ± 1.12	4.36 ± 1.32	0.382
HDL	1.12 ± 0.29	0.94 ± 0.28	0.001
LDL	2.75 ± 0.85	2.69 ± 1.05	0.696
Urea	6.99 ± 15.35	5.74 ± 2.36	0.576
Crea	71.31 ± 78.36	71.78 ± 28.98	0.968
ALB	42.93 ± 5.01	42.06 ± 4.62	0.316
AST	90.90 ± 627.72	27.32 ± 23.09	0.485
ALT	79.61 ± 484.66	36.20 ± 19.87	0.537

### 2.6 Circ‐PIAS1‐5 Serves as a Sponge for miR‐219a‐2‐3p to Inhibit Lipid Accumulation in Foam Cells

**Figure 6:**
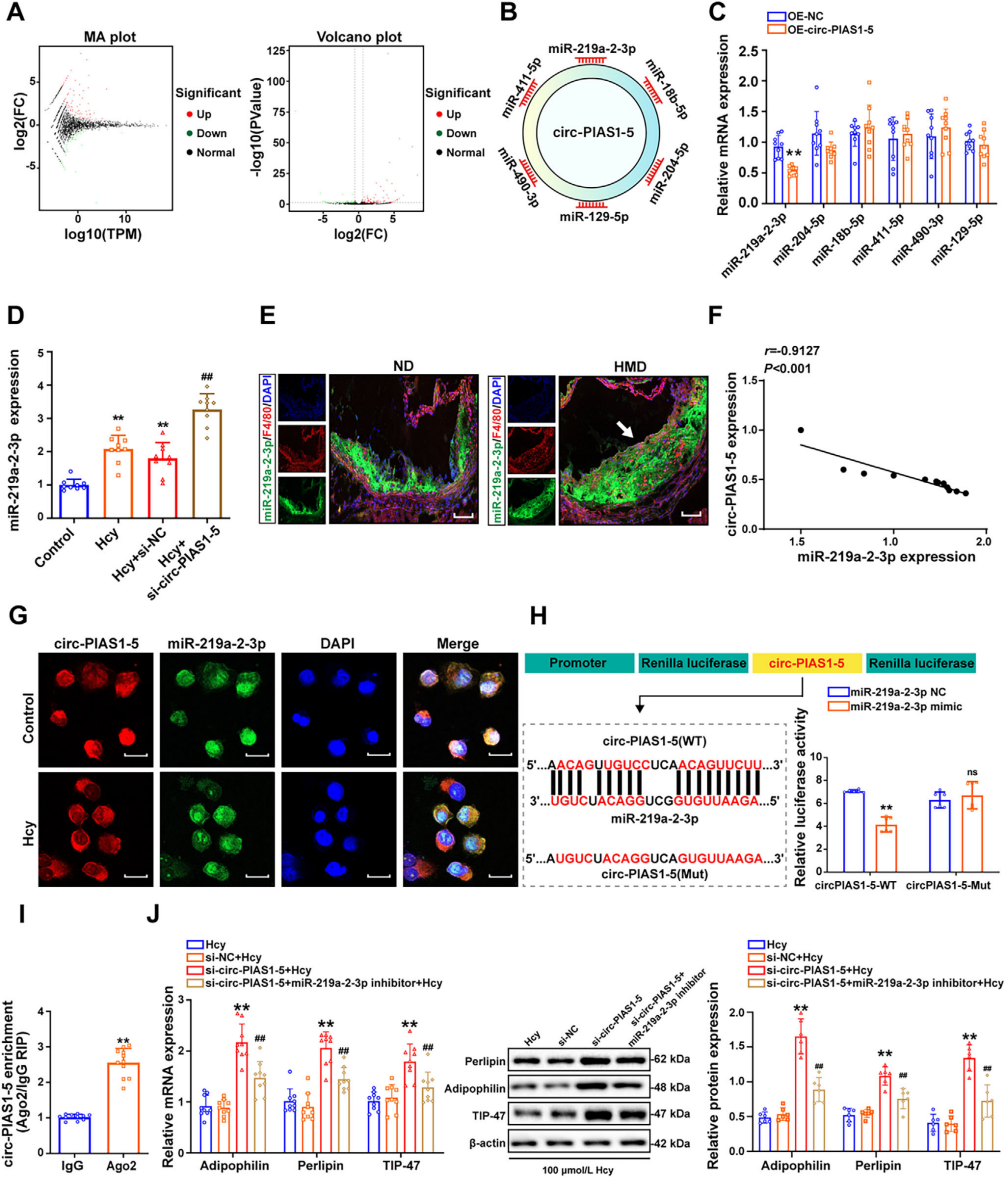
Circ‐PIAS1‐5 serves as a sponge for miR‐219a‐2‐3p and regulates lipid accumulation in atherosclerosis. A) N‐methyladenosine (mA) map and volcano plot showing the miRNAs that were differentially expressed (fold change ≥ 1.5 and *p* < 0.05) in foam cells treated with Hcy. The red dots and green dots indicate the upregulated and downregulated miRNAs, respectively. B) A schematic model showing that miR‐219a‐2‐3p, miR‐204‐5p, miR‐18b‐5p, miR‐490‐3p, miR‐129‐5p, and miR‐411‐5p have putative binding sites with circ‐PIAS1‐5 according to RNA‐hybrid miRNA target prediction tools. C) Relative expression of miR‐219a‐2‐3p, miR‐204‐5p, miR‐18b‐5p, miR‐490‐3p, miR‐129‐5p, and miR‐411‐5p was measured by RT‐PCR in foam cells transfected with OE‐circ‐PIAS1‐5. D) Relative expression of miR‐219a‐2‐3 was detected by qRT‐PCR in foam cells transfected with si‐circ‐PIAS1‐5. E) The expression of miR‐219a‐2‐3p (green) and F4/80 (red) in the aortas of ApoE^−/−^ mice fed high methionine concentrations was detected via RNA‐FISH. The nuclei were stained with DAPI (blue) (scale bar = 10 µm). F) Pearson's correlation analysis of the expression of circ‐PIAS1‐5 with that of miR‐219a‐2‐3p in ApoE^−/−^ mice. G) Co‐localization of circ‐PIAS1‐5 (red) and miR‐219a‐2‐3p (green) was observed via RNA‐FISH in foam cells treated with Hcy. The nuclei were stained with DAPI (blue) (scale bar = 10 µm). H) Schematic diagram depicting the constructed firefly luciferase reporter system and the predicted binding sites of miR‐219a‐2‐3p in the circ‐PIAS1‐5 sequence. The relative luciferase activities were detected in foam cells after co‐transfection of miR‐219a‐2‐3p mimics with circ‐PIAS1‐5‐WT or circ‐PIAS1‐5‐Mut. I) An RIP assay was performed using an Ago2 antibody in foam cells, and the expression of circ‐PIAS1‐5 was detected by qRT‐PCR. J) Perilipin, adipophilin, and TIP47 expression was determined by qRT‐PCR and western blot analysis in foam cells transfected with si‐circ‐PIAS1‐5 and the miR‐219a‐2‐3p inhibitor. The data are presented as the mean ± SD. ^*^ *p* < 0.05, ^**^ *p* < 0.01, compared with the OE‐NC group, the control group, the IgG group or the si‐NC + Hcy group; ^##^ *p* < 0.01, compared with the si‐circ‐PIAS1‐5 + Hcy group.

Because circRNAs function as sponges of miRNAs, we performed miRNA‐seq profiling in foam cells treated with Hcy to identify miRNAs related to circ‐PIAS1‐5. The results revealed 86 upregulated miRNAs and 36 downregulated miRNAs in foam cells after Hcy treatment (**Figure** **6**A). Among them, miR‐219a‐2‐3p, miR‐204‐5p, miR‐18b‐5p, miR‐490‐3p, miR‐129‐5p, and miR‐411‐5p have putative binding sites for circ‐PIAS1‐5 according to RNA‐hybrid miRNA target prediction tools (Figure 6B). However, among the five differentially expressed miRNAs, only miR‐219a‐2‐3p was significantly decreased after the foam cells were transfected with OE‐circ‐PIAS1‐5, and miR‐219a‐2‐3p expression was reversed after the foam cells were transfected with si‐circ‐PIAS1‐5 (Figure 6C,D). Furthermore, the expression of miR‐219a‐2‐3p in ApoE^−/−^ mice was significantly increased in the aortas of ApoE^−/−^ mice fed high methionine concentrations (Figure 6E). Pearson's correlation analysis also revealed that miR‐219a‐2‐3p expression was negatively correlated with circ‐PIAS1‐5 expression in ApoE^−/−^ mice (Figure 6F), indicating a possible interaction between miR‐219a‐2‐3p and circ‐PIAS1‐5. Additionally, RNA‐FISH assays revealed that both circ‐PIAS1‐5 and miR‐219a‐2‐3p were predominantly located in the cytoplasm of foam cells treated with Hcy (Figure 6G). The potential binding site in circ‐PIAS1‐5 for its binding with miR‐219a‐2‐3p, which was identified via the miRanda database, was mutated. The results showed that the miR‐219a‐2‐3p mimic notably suppressed the luciferase activity of the circ‐PIAS1‐5 wild‐type reporter (circ‐PIAS1‐5‐WT) but not the mutant reporter (circ‐PIAS1‐5‐Mut) (Figure 6H). The Ago2 protein acts as a core component of the RNA‐induced silencing complex that binds miRNA complexes to target mRNAs, which is an indicator protein for circRNAs to act as sponges. The results of the RNA immunoprecipitation (RIP) assay revealed that, compared with control Immunoglobulin G (IgG), circ‐PIAS1‐5 was specifically enriched in beads containing the Ago2 antibody, suggesting the occupancy of Ago2 in the region of circ‐PIAS1‐5 (Figure 6I). As expected, qRT‐PCR and western blot assays demonstrated that knockdown of circ‐PIAS1‐5 increased the expression of perilipin, adipophilin, and TIP47 in foam cells treated with Hcy, whereas inhibition of miR‐219a‐2‐3p reversed the expression of perilipin, adipophilin, and TIP47 in foam cells transfected with si‐circ‐PIAS1‐5 (Figure 6J). Taken together, these results demonstrated that circ‐PIAS1‐5 could act as an miR‐219a‐2‐3p sponge in atherosclerosis induced by Hcy.

### 2.7 Circ‐PIAS1‐5 Activates the AMP‐Activated Protein Kinase Pathway by Regulating TEAD1 and Contributes to Atherosclerosis

**Figure 7:**
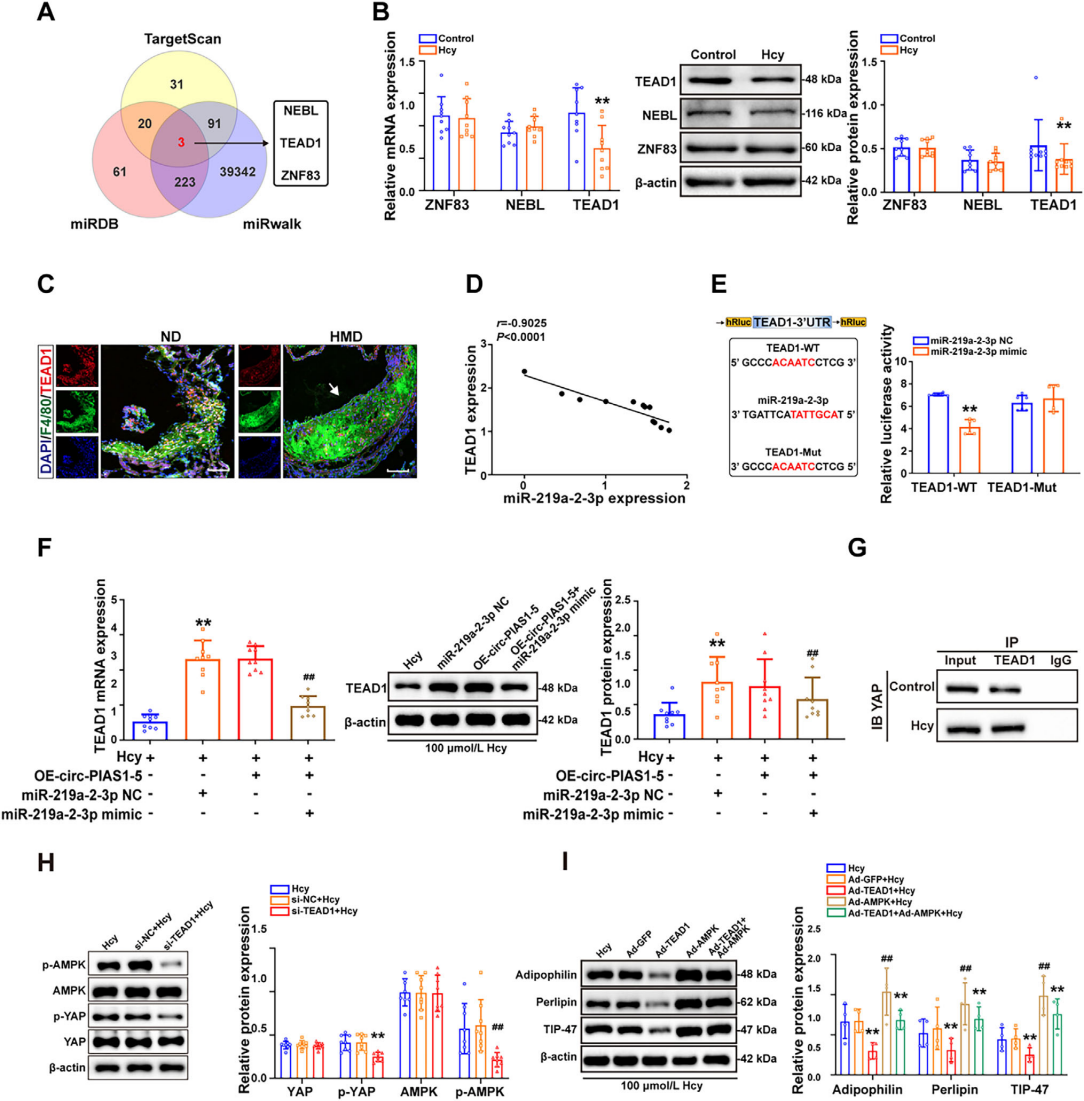
Circ‐PIAS1‐5 activates the AMP‐activated protein kinase pathway via TEAD1, leading to atherosclerosis. A) Venn diagram illustrating the overlap of potential target genes of miR‐219a‐2‐3p predicted via miRDB, miRTarBase, and TargetScan. B) qRT‐PCR and western blot analysis were performed to detect the expression of TEAD1, NEBL, and ZNF83 in foam cells treated with Hcy. C) Representative immunofluorescence images and quantification of TEAD1 (red) co‐localization with F4/80 (green) in the aortas of ApoE^−/−^ mice fed a high‐methionine diet. Nuclei were stained with DAPI (blue) (scale bar = 20 µm). D) The correlation between TEAD1 and miR‐219a‐2‐3p expressions was evaluated via Pearson's correlation analysis. E) Luciferase reporters for TEAD1 wild‐type (TEAD1‐WT) and mutant (TEAD1‐Mut) genes were constructed, and the relative luciferase activities were measured via a luciferase reporter assay in 293T cells co‐transfected with TEAD1‐WT or TEAD1‐Mut and an miR‐219a‐2‐3p NC or an miR‐219a‐2‐3p mimic. F) TEAD1 expression in foam cells co‐transfected with OE‐circ‐PIAS1‐5 and the miR‐219a‐2‐3p mimic in the presence of Hcy was determined via qRT‐PCR and western blot analysis. G) Foam cells were treated with Hcy, and total cell lysates were subsequently immunoprecipitated with TEAD1 antibodies or control IgG and subjected to western blot analysis with anti‐YAP antibodies. H) The expressions of yes‐associated protein (YAP), phosphorylated YAP (p‐YAP), AMP‐activated protein kinase, and phosphorylated AMP (p‐AMP)‐activated protein kinase were detected by western blot in foam cells transfected with TEAD1 small interfering RNA (si‐TEAD1) in the presence of Hcy. I) The expression of perilipin, adipophilin, and TIP47 in foam cells co‐transfected with si‐TEAD1 and adenovirus overexpressing MAPK (Ad‐MAPK) in the presence of Hcy was detected via western blotting. The data are presented as the mean ± SD. ^**^ *p* < 0.01, compared with the control group, the Hcy group, the miR‐219a‐2‐3p NC group, or the Ad‐GFP + Hcy group. ^##^ *p* < 0.01, compared with the si‐NC + Hcy group or the Hcy group.

To determine how circ‐PIAS1‐5 contributes to the pathogenesis of atherosclerosis as a sponge of miR‐219a‐2‐3p, TEAD1, NEBL, and ZNF83 were identified as potential targets of miR‐219a‐2‐3p via bioinformatics software (miRDB, miRTarBase, and TargetScan databases) (**Figure** **7**A). The qRT‐PCR and western blot results confirmed that TEAD1, but not the NEBL and ZNF83 targets, was downregulated in foam cells treated with Hcy (Figure 7B), which was further confirmed by co‐localization analysis of TEAD1 with F4/80 in the atherosclerotic plaques of ApoE^−/−^ mice fed a high‐methionine diet (Figure 7C). The correlation between TEAD1 and miR‐219a‐2‐3p expressions revealed that TEAD1 expression was inversely associated with miR‐219a‐2‐3p expression in the aortas of ApoE^−/−^ mice (Figure 7D). Furthermore, we applied a dual‐luciferase reporter assay to determine whether miR‐219a‐2‐3p could directly bind TEAD1. Transfection of the miR‐219a‐2‐3p mimic significantly reduced the luciferase activity of wild‐type TEAD1 (WT‐TEAD1) but not mutant TEAD1 (Mut‐TEAD1) (Figure 7E), suggesting that TEAD1 is a functional target gene of miR‐219a‐2‐3p. In addition, an miR‐219a‐2‐3p mimic and OE‐circ‐PIAS1‐5 were co‐transfected into foam cells to validate the effects of the circ‐PIAS1‐5/miR‐219a‐2‐3p axis on TEAD1 expression. The results also revealed that overexpression of circ‐PIAS1‐5 notably increased the expression of TEAD1, which was reversed by the miR‐219a‐2‐3p mimic (Figure 7F). TETE1 has been reported to regulate adenosine 5‘‐monophosphate (AMP)‐activated protein kinase activation through yes‐associated protein (YAP). We detected the interaction between endogenous YAP and TEAD1 in foam cells treated with Hcy via Co‐Immunoprecipitation (CO‐IP). The results showed that YAP could interact with TEAD1 (Figure 7G). Furthermore, we knocked down and overexpressed TEAD1 in foam cells via infection with si‐TEAD1 and Ad‐TEAD1, respectively, to verify whether TEAD1 regulates lipid accumulation via activated YAP/AMP‐activated protein kinase signaling (Figure S5, Supporting Information). The results revealed that the phosphorylation levels of YAP (p‐YAP) and AMP (p‐AMP)‐activated protein kinase were decreased in foam cells transfected with si‐TEAD1 (Figure 7H). Moreover, western blot results revealed that TEAD1 overexpression decreased the levels of perilipin, adipophilin, and TIP47, which were restored when AMP‐activated protein kinase was overexpressed (Figure 7I). Taken together, these results demonstrated that circ‐PIAS1‐5 activates the AMP‐activated protein kinase pathway by regulating TEAD1 and contributes to atherosclerosis in ApoE^−/−^ mice.

### 2.8 YTHDC1 Mediates the Nuclear Export of circ‐PIAS1‐5, Leading to Lipid Accumulation Induced by Hcy

**Figure 8:**
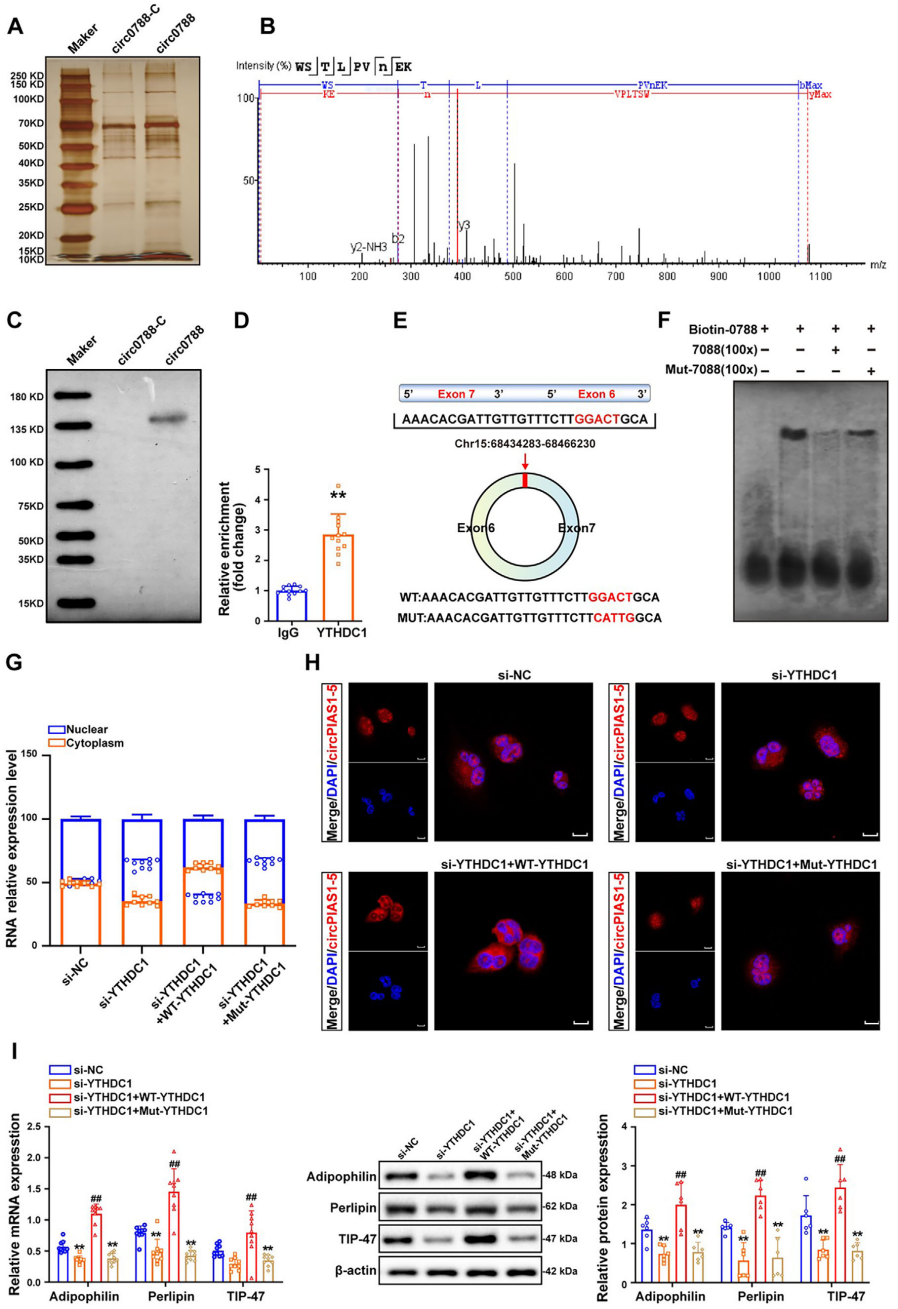
Hcy promotes the nuclear translocation of circ‐PIAS1‐5 via YTHDC1, contributing to lipid accumulation. A) Biotin‐labeled linear or circ‐PIAS1‐5 RNA transcripts were incubated with foam cell lysates, separated by SDS‐PAGE, and visualized via silver staining. Protein bands in different regions from each lane (indicated by rectangular boxes) were subjected to mass spectrometry to identify circ‐PIAS1‐5‐interacting proteins. B) Identification of the expression peak of the YTHDC1 protein, and circ‐PIAS1‐5 binding was detected via mass spectrometry (MS) analysis. C) Western blot analysis showing the proteins pulled down from the lysates of foam cells by biotin‐labeled linear or circ‐PIAS1‐5 probes. D) An RNA immunoprecipitation (RIP) assay was performed using YTHDC1 or IgG antibodies, followed by qRT‐PCR assay for circ‐PIAS1‐5 expression in foam cells treated with Hcy. IgG served as an antibody control. E) Schematic illustration showing the YTHDC1 and circ‐PIAS1‐5 binding sites located at the exon 5–exon 4 junction site of circ‐PIAS1‐5. F) RNA‐EMSA was used to detect the binding ability of purified YTHDC1 with biotin‐labeled circ‐PIAS1‐5 in foam cells transfected with the wild‐type circ‐PIAS1‐5 sequence (WT‐circ‐PIAS1‐5) or a point mutation (Mut‐PIAS1‐5) in the binding sequence of circ‐PIAS1‐5 and YTHDC1 in the presence of Hcy. G) Nuclear and cytoplasmic separation assays were used to detect the expression of circ‐PIAS1‐5 in foam cells after transfection with YTHDC1 small interfering RNA (si‐YTHDC1) and/or YTHDC1 wild type (WT‐YTHDC1)/mutant YTHDC1 (Mut‐YTHDC1) in the presence of Hcy. H) RNA‐FISH was used to detect the location of circ‐PIAS1‐5 in foam cells after transfection with si‐YTHDC1 and/or WT‐YTHDC1/Mut‐YTHDC1 in the presence of Hcy (scale bar = 10 µm). I) The expressions of perilipin, adipophilin, and TIP‐47 in foam cells were measured by qRT‐PCR and western blot analysis after transfection with si‐YTHDC1 and/or WT‐YTHDC1/Mut‐YTHDC1 in the presence of Hcy. The data are presented as the mean ± SD. ^**^ *p* < 0.01, compared with the lgG group, the Hcy group, or the si‐NC group. ^##^ *p* < 0.01, compared with the si‐YTHDC1 group.

To explore the proteins that directly bind to circ‐PIAS1‐5 peptides in foam cells, we performed biotin‐labeled linear or circ‐PIAS1‐5 pulldown followed by mass spectrometry (MS) analysis of the RNA‐associated protein complex in foam cells. Silver gel staining revealed that several enriched bands from 65 to 75 kDa were significantly enriched in biotin‐labeled circ‐PIAS1‐5 compared with biotin‐labeled linear bands in foam cells (**Figure** **8**A). On the basis of the MS analysis, we selected the YTHDC1 proteins with the highest score and matches for further validation by RNA pulldown followed by MS analysis (Figure 8B). As determined by an RNA pulldown assay, YTHDC1 was enriched in biotin‐labeled circ‐PIAS1‐5 compared with biotin‐labeled linear circ‐PIAS1‐5 in foam cells (Figure 8C). RIP in combination with qRT‐PCR analysis confirmed the specific interaction between circ‐PIAS1‐5 and YTHDC1 in foam cells. Consistent with these findings, we observed significantly greater enrichment of circ‐PIAS1‐5 with the YTHDC1 antibody than with the IgG antibody, suggesting the increased interaction of circ‐PIAS1‐5 with YTHDC1 in foam cells (Figure 8D). We subsequently used circScan34 to predict the binding sites of YTHDC1 and circ‐PIAS1‐5, and the results revealed that YTHDC1 interacted with circ‐PIAS1‐5 at the exon 5–exon 4 junction site of circ‐PIAS1‐5 (hg19, chr5: 6623326–6625782) (Figure 8E). An RNA electrophoretic mobility shift assay (RNA–EMSA followed by western blot revealed that the ability of YTHDC1 to interact with circ‐PIAS1‐5 decreased after the binding sites of YTHDC1 and circ‐PIAS1‐5 were mutated (Figure 8F). YTHDC1 is a new m^6^A “reader” that is implicated in regulating the redistribution of m^6^A‐marked transcripts from the nucleus to the cytoplasm, back‐splicing, and co‐transcriptional interplay. Interestingly, nuclear and cytoplasmic fractionation experiments revealed that circ‐PIAS1‐5 shuttled from the cytosol to the nucleus in foam cells transfected with YTHDC1 small interfering RNA (si‐YTHDC1) but decreased circ‐PIAS1‐5 cytosolic shuttling after transfection with YTHDC1 wild type (WT‐YTHDC1) or mutant YTHDC1 (Mut‐YTHDC1) had no significant effect (Figure 8G). RNA‐FISH confirmed the cytosolic accumulation of circ‐PIAS1‐5 in foam cells transfected with si‐YTHDC1 (Figure 8H). Importantly, the expressions of the lipid‐binding proteins perilipin, adipophilin, and TIP47 were significantly decreased in foam cells transfected with si‐YTHDC1 but was restored by WT‐YTHDC1 transfection (Figure 8I). Collectively, these results indicate that YTHDC1 combined with circ‐PIAS1‐5 to facilitate nuclear translocation contributes to lipid accumulation in foam cells.

### 2.9 Hcy Induces m^6^A Modification of Circ‐PIAS1‐5 to Regulate Its Function as an miRNA Sponge through YTHDC1 in Foam Cells

**Figure 9:**
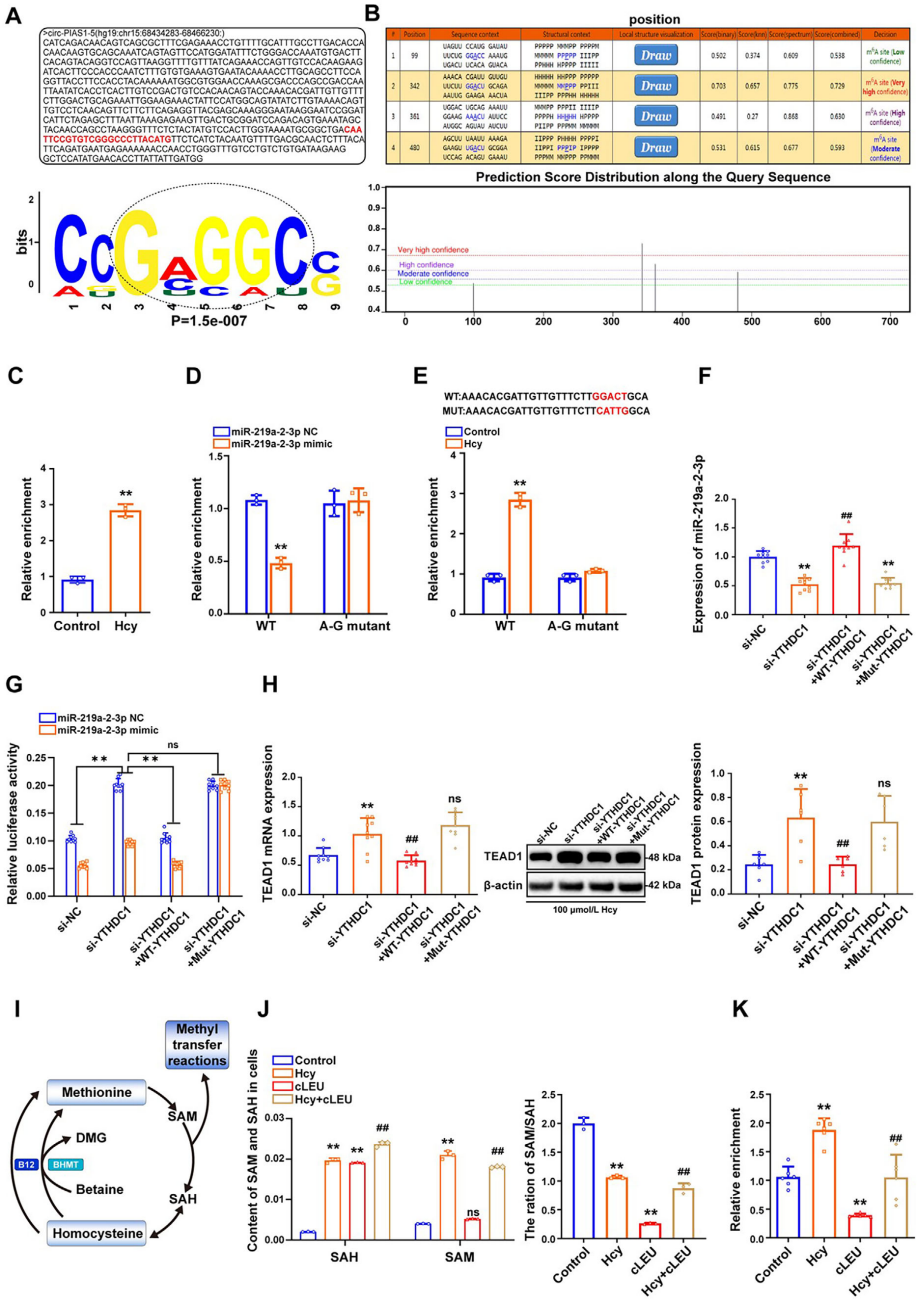
m^6^A modification of circ‐PIAS1‐5 regulates its function as an endogenous RNA through YTHDC1 in foam cells. A) Sequence motifs of the m^6^A‐containing peak regions of circ‐PIAS1‐5 were identified via HOMER analysis. B) Prediction score distribution along m^6^A modification sites via SRAMP software. C) MeRIP‐PCR analysis of m^6^A enrichment of circ‐PIAS1‐5 in foam cells treated with Hcy. Anti‐IgG antibodies were used as a control. D) m^6^A enrichment of circ‐PIAS1‐5 was detected by MeRIP‐PCR in foam cells transfected with WT‐circ‐PIAS1‐5 or m^6^A site mutants (Mut‐circ‐PIAS1‐5) in the presence of Hcy. E) The relative luciferase activity of circ‐PIAS1‐5 was detected by luciferase reporter assays in HEK293T cells co‐transfected with the miR‐219a‐2‐3p mimic and WT‐circ‐PIAS1‐5 or Mut‐circ‐PIAS1‐5. The firefly luciferase activity was normalized to that of the control renilla. F) The expression of miR‐219a‐2‐3p was detected by qRT‐PCR in foam cells co‐transfected with YTHDC1 small interfering RNA (si‐YTHDC1) and WT‐YTHDC1 or Mut‐YTHDC1. G) The relative luciferase activity of circ‐PIAS1‐5 was detected by luciferase reporter assays in foam cells co‐transfected with si‐YTHDC1 and WT‐YTHDC1 or Mut‐YTHDC1. H) qRT‐PCR and western blot analysis of TEAD1 expression in foam cells co‐transfected with si‐YTHDC1 and WT‐YTHDC1 or Mut‐YTHDC1 in the presence of Hcy. I) Schematic diagram of the Hcy–methionine cycle. Hcy is biosynthesized from methionine by S‐adenosylmethionine (SAM) and S‐adenosylhomocysteine hydrolase (SAH). Hcy may be remethylated to methionine‐by‐methionine synthase, which requires folate, vitamin B_12_, and betaine homocysteine S‐methyltransferase (BHMT). SAM is transformed into SAH by donating its methyl group to the substrates of methylation reactions. J) High‐performance liquid chromatography (HPLC) was performed to measure the intracellular SAM and SAH levels in foam cells treated with cLEU (an inhibitor of SAM transferase) in the presence of Hcy. K) MeRIP‐PCR was used to detect the m^6^A enrichment of circ‐PIAS1‐5 in foam cells treated with eLEU in the presence of Hcy. The data are presented as the mean ± SD. ^**^ *p* < 0.01, compared with the control group, the miR‐219a‐2‐3p NC group, or the si‐NC group. ^##^ *p* < 0.01, compared with the si‐YTHDC1 group or the Hcy group.

**Figure 10:**
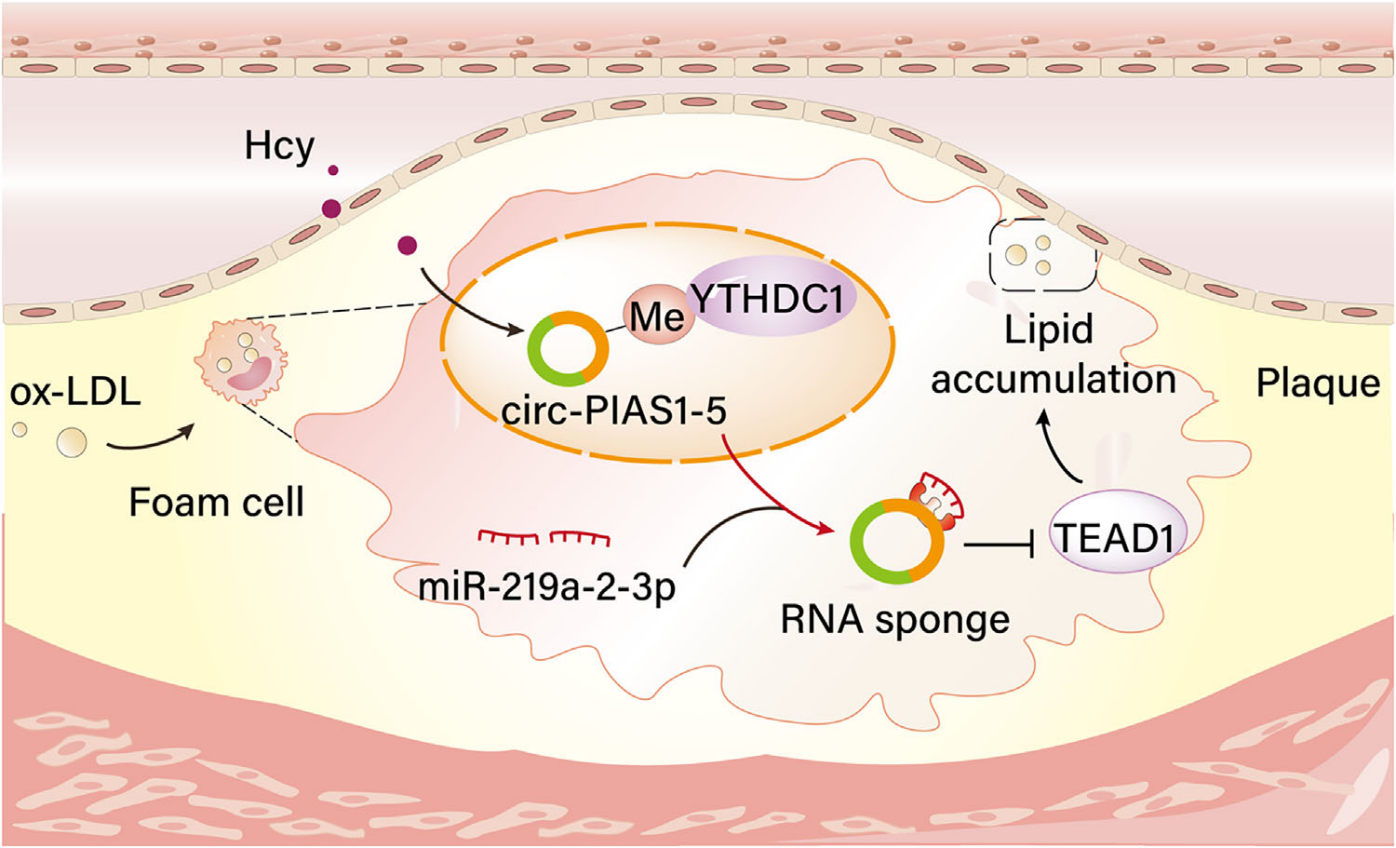
Schematic representation of a model for the major molecular mechanisms by which Hcy accelerates the nuclear export of circ‐PIAS1‐5, which regulates atherosclerosis by acting as a competing endogenous RNA for miR‐219a‐2‐3p. Hcy enrichment of circ‐PIAS1‐5 leads to YTHDC1 binding to circ‐PIAS1‐5 and promotes its intracellular localization from the nucleus to the cytoplasm, which contributes to circ‐PIAS1‐5 acting as a sponge of miR‐219a‐5p to promote lipid accumulation in foam cells in Hcy‐induced atherosclerosis through regulating TEAD1.

To investigate whether Hcy regulates the m^6^A modification of circ‐PIAS1‐5 in foam cells, we searched for consensus motifs deposited in the region surrounding the m^6^A peak of circ‐PIAS1‐5 via the HOMER package and found a classical AAACH motif structure for m^6^A methylation at the circ‐PIAS1‐5 sequence (**Figure** **9**A). Moreover, two high‐confidence m^6^A sites close to the junction region of circ‐PIAS1‐5 were identified via the SRAMP database (Figure 9B). Consistent with the bioinformatics analysis results, methylated RNA immunoprecipitation (MeRIP) combined with qRT‐PCR revealed that circ‐PIAS1‐5 was abundantly enriched by the m^6^A antibody compared with the negative control IgG antibody in foam cells treated with Hcy (Figure 9C). However, lower m^6^A enrichment was detected in foam cells transfected with m^6^A site mutants (Mut‐circ‐PIAS1‐5) (Figure 9D), which confirmed that circ‐PIAS1‐5 is modulated by m^6^A in foam cells treated with Hcy. To verify the effect of circ‐PIAS1‐5 m^6^A modification on its function as an endogenous RNA for miR‐219a‐2‐3p, we performed luciferase reporter assays in HEK293T cells after transfection with the miR‐219a‐2‐3p mimic combined with the circ‐PIAS1‐5 wild type (WT‐circ‐PIAS1‐5) and m^6^A site mutant type (Mut‐circ‐PIAS1‐5). As shown in Figure 9E, transfection of the miR‐219a‐2‐3p mimic significantly increased the luciferase activity of WT‐circ‐PIAS1‐5 but not Mut‐circ‐PIAS1‐5. Foam cells were subsequently co‐transfected with si‐YTHDC1 and WT‐YTHDC1 or m^6^A‐binding‐defective YTHDC1 (Mut‐YTHDC1). The results revealed that the expression of miR‐219a‐2‐3p was decreased in foam cells transfected with si‐YTHDC1, and WT‐YTHDC1 transfection reversed the downregulation of miR‐219a‐2‐3p expression caused by si‐YTHDC1, whereas transfection with Mut‐YTHDC1 did not achieve this effect (Figure 9F). In addition, luciferase reporter assays revealed that the effect of miR‐219a‐2‐3p on circ‐PIAS1‐5 was abolished upon mutation of YTHDC1 (Figure 9G). Moreover, the expression of TEAD1 was increased in foam cells transfected with WT‐YTHDC1, whereas Mut‐YTHDC1 transfection restored the expression of TEAD1 (Figure 9H), suggesting that YTHDC1 binds to circ‐PIAS1‐5 via recognition of m^6^A modification to modulate its function as an endogenous RNA for miR‐219a‐2‐3p. As the direct metabolite of methionine, S‐adenosylmethionine (SAM) is the universal methyl donor for cellular methylation (Figure 9I). Interestingly, SAM and S‐adenosylhomocysteine (SAH) levels were significantly increased in foam cells treated with Hcy, whereas cLEU (an inhibitor of SAM transferase) treatment increased the SAM and SAH levels in foam cells treated with Hcy (Figure 9J). The MeRIP results revealed that cLEU reversed the m^6^A enrichment of circ‐PIAS1‐5 in foam cells treated with Hcy (Figure 9K). Taken together, these results provide strong evidence that Hcy induces m^6^A modification of circ‐PIAS1‐5 to regulate its function through YTHDC1 in foam cells (**Figure** **10**).

## 3 Discussion

Atherosclerosis, a chronic disease characterized by fibrofatty lesions formed in the artery walls, is considered a major cause of various CVDs.^[^ ^21^ ^]^ Substantial evidence suggests that disorders of lipid metabolism, such as excessive uptake of ox‐LDL and impaired cholesterol outflow, accelerate the progression of atherosclerosis.^[^ ^22^ ^]^ Hcy is a sulfhydryl‐containing amino acid synthesized as an intermediate metabolite in the methionine cycle and is considered an independent risk factor for atherosclerosis.^[^ ^23^ ^]^ Hcy has been reported to accelerate lipid accumulation to promote the progression of atherosclerotic lesions.^[^ ^24^ ^]^ However, the underlying mechanism of lipid metabolism disorders in foam cells during the development of atherosclerosis induced by Hcy requires further exploration. In the present study, we identified a novel lipid metabolism‐associated circRNA, circ‐PIAS1‐5, whose expression pattern, potential diagnostic value, and prognostic significance were assessed in disorders of lipid metabolism in foam cells from patients with atherosclerosis. Mechanistically, we revealed that nuclear export of circ‐PIAS1‐5 is mediated by YTHDC1 in an m^6^A‐dependent manner. Importantly, cytoplasmic expression of circ‐PIAS1‐5 alleviates miR‐219a‐2‐3p expression, causing downregulation of TEAD1 in foam cells, which consequently regulates the progression of atherosclerosis induced by Hcy.

CircRNAs are novel noncoding RNAs with closed loop structures that circularize from exons, or some have lasso structures that circularize from introns.^[^ ^25^ ^]^ Because circRNAs do not contain 5′ or 3′ ends, they are more stably expressed and are not easily degraded.^[^ ^26^ ^]^ Recent studies have shown that circRNAs are abnormally expressed in different diseases and play a regulatory role in different diseases.^[^ ^27^, ^28^ ^]^ For example, research on the roles of circRNAs in atherosclerosis has progressed rapidly, and they have been implicated in the pathophysiological processes underlying the development of atherosclerosis, including changes in the functions of endothelial cells, vascular smooth muscle cells, and macrophages.^[^ ^29^ ^]^ Hence, the potential of circRNAs for atherosclerosis treatment has been confirmed by numerous studies. In this study, we first identified a novel circular RNA through circRNA microarray analysis and determined that circ‐PIAS1‐5 was downregulated in atherosclerotic plaques and foam cells treated with Hcy. Subsequently, functional experiments revealed that circ‐PIAS1‐5 reversed lipid accumulation in foam cells and plaque formation. These findings provide a perspective on the diagnosis and treatment of atherosclerotic plaque formation in circ‐PIAS1‐5. As atherosclerosis underlies the bulk of cardiovascular disorders, new and highly sensitive/convenient diagnostic biomarkers for atherosclerosis development are needed, which may aid in monitoring disease progression and therefore be highly valuable in terms of human health, as well as social economics. CircRNAs are novel therapeutic targets for various diseases, and they can regulate cellular pathways and modulate diverse diseases.^[^ ^30^ ^]^ The structural properties of circRNAs include their presence in body fluids such as plasma and saliva, which allows their application as potential clinical biomarkers.^[^ ^31^ ^]^ In this study, clinical data revealed that circ‐PIAS1‐5 expression in the blood was decreased in patients with atherosclerosis complicated by HHcy and was significantly correlated with Hcy and HDL concentrations but not with TC, TG, LDL, urea, Crea, ALB, AST, or ALT concentrations. Importantly, downregulation of circ‐PIAS1‐5 was implicated as an independent prognostic marker for patients with atherosclerosis complicated by HHcy. These findings provide a basis for the application of circ‐PIAS1‐5 as a novel diagnostic biomarker of atherosclerosis, which may serve as a therapeutic target for managing the progression of atherosclerotic disease in HHcy patients. However, the mechanism of circ‐PIAS1‐5 in Hcy‐induced atherosclerosis is poorly understood.

CircRNAs are considered promising candidates for the regulation of gene expression because they act as cytoplasmic miRNA sponges and transcriptional or post‐transcriptional regulators.^[^ ^32^ ^]^ They can compete with linear RNA by affecting the accumulation of full‐length mRNA. Notably, many validated competing endogenous RNA networks of gene expression regulators have been demonstrated to participate in the initiation and progression of various diseases.^[^ ^33^ ^]^ However, the molecular mechanism underlying their role as miRNA sponges in disorders of lipid metabolism in foam cells has not been elucidated clearly. Here, our results revealed that circ‐PIAS1‐5 modulates TEAD1 expression by sponging miR‐219a‐2‐3p, thus inhibiting lipid accumulation in foam cells. The TEAD transcription factor family is a component of the Hippo signaling pathway, the inactivation of which has been implicated in cell replication, growth, and development. In this study, we found that the interaction between YAP and TEAD1 activated the YAP and AMP‐activated protein kinase pathways, contributing to lipid accumulation in foam cells. Moreover, a significant negative correlation between circ‐PIAS1‐5 and miR‐219a‐2‐3p suggested that detectable cellular miR‐219a‐2‐3p increased after being sponged. These results are consistent with those of previous studies, suggesting that competing endogenous RNA networks affect atherogenesis by modulating lipid accumulation. Although our findings revealed that circ‐PIAS1‐5 acts as an miRNA sponge in atherosclerotic plaque formation, there may be other roles for these circRNAs that should be further evaluated.

m^6^A modification is an abundant co‐transcriptional modification of mRNAs and ncRNAs, including circRNAs in mammals.^[^ ^34^ ^]^ m^6^A modification can affect many functions, such as stability, splicing, export, translation, and decay. However, very few studies on the effects of m^6^A modification on the biology of cellular circRNAs have been reported.^[^ ^35^ ^]^ In our study, Hcy promoted circ‐PIAS1‐5 nuclear translocation. Therefore, investigating the underlying mechanism that regulates the export of circRNAs from the nucleus to the cytoplasm is critical.^[^ ^36^ ^]^ N‐methyladenosine readers play important roles in mRNA functions and metabolism, and YTH domain‐containing proteins participate extensively in post‐transcriptional regulation by regulating the splicing, translation, localization, and lifetime of RNAs by targeting different complexes to specific sites via direct binding to m^6^A modification sites.^[^ ^37^ ^]^ It has been reported that there are five YTH domain‐containing proteins in humans. Among them, YTHDC1 is a nuclear protein involved in gene splicing.^[^ ^38^ ^]^ Our results strongly suggest that markedly increased YTHDC1 has a distinct function in promoting the nuclear export of m^6^A‐modified circ‐PIAS1‐5. We discovered that high m^6^A modification of circ‐PIAS1‐5 and the circ‐PIAS1‐5 methylation site could bind to YTHDC1 and export it from the nucleus to the cytoplasm in a m^6^A‐dependent manner, supporting the emergence of m^6^A as a potential selective signal for the metabolism of mammalian circRNAs.

In summary, we constructed a circRNA‐centric noncoding regulatory RNA network via genome‐wide analysis of the mRNA, circRNA, and miRNA expression profiles of Hcy‐induced atherosclerosis. The existence and relevant function of circ‐PIAS1‐5 in Hcy‐induced atherosclerosis were determined. We identified a circRNA, circ‐PIAS1‐5, that was revealed to inhibit lipid deposition in foam cells in Hcy‐induced atherosclerosis. More importantly, we propose that YTHDC1 promotes circ‐PIAS1‐5 nuclear output to affect the ability of circ‐PIAS1‐5 to act as an endogenous RNA for miR‐219a‐2‐3p. The circ‐PIAS1‐5 expression level was decreased and had satisfactory sensitivity and specificity in patients with atherosclerosis complicated by HHcy. This study provides a better understanding of the molecular mechanism of Hcy‐induced atherosclerosis and will allow proper early clinical application of these findings for diagnosis and precise therapy. Therefore, innovations in epigenetic therapies targeting atherosclerosis‐causing circRNAs are expected in the future.

## 4 Experimental Section

### Study Samples

A total of 140 individuals, including 70 healthy controls and 70 patients with atherosclerosis concomitant with HHcy, were recruited from the General Hospital of Ningxia Medical University between January 2019 and May 2022. The classification of HHcy was based on the final diagnosis at discharge. Individuals with congenital heart disease, rheumatic valvular disease, cardiomyopathy, stroke, diabetes mellitus, malignant tumors, acute or chronic infections, or severe liver or kidney dysfunction were excluded from the study. The clinical information of the healthy controls and atherosclerotic patients with HHcy is summarized in Table 1. Written informed consent was obtained from all patients or their families under the Declaration of Helsinki, and the study was approved by the Ethics Committee of the General Hospital of Ningxia Medical University (2020[914]).

### Animal Treatment

Six‐week‐old male ApoE^−/−^ mice were purchased from the Laboratory Animal Center of Peking University Health Science Center (Beijing, China). All animals received humane care in compliance with the Institutional Authority for Laboratory Animal Care of Ningxia Medical University in accordance with the Guide for the Care and Use of Laboratory Animals published by the United States National Institutes of Health. They were housed individually in a climate‐controlled room (24 °C) with a 12/12 h light/dark cycle and provided food and water ad libitum. After 1 week of acclimatization, the mice were randomly divided into two groups and fed a chow diet (ND) or a chow diet plus 1.7% methionine (HMD) for 16 weeks. In addition, the experimental mice were injected with AAV‐circ‐PIAS1‐5 or a mock vector with AAV‐circ‐NC (Shanghai GenePharma Co., Ltd.) at a dose of 100 µL once a week via the tail vein after receiving a chow diet plus 1.7% methionine for 13 weeks. At termination, the mice were anaesthetized with isoflurane before blood and tissue samples were obtained for further analysis.

### Tissue Preparation and Atherosclerotic Lesion Evaluation in ApoE^−/−^ Mice

The mice were anaesthetized with isoflurane and then fixed at the B‐ultrasound station to measure the ascending main blood flow of the aortic arch and the intima thickness of the aortic arch. After blood sampling, the mice were sacrificed, and their hearts were flushed with saline. The aortas were embedded in optimal cutting temperature (OCT) compound and snap frozen in liquid N_2_. The inferior vena cava was cut to allow the perfusate to exit. Frozen sections of 4 µm thickness were taken in the region of the proximal aorta, starting from the end of the aortic sinus and 300 mm distal. The sections were stained with HE and Oil Red O. Quantitative analysis of lipid‐stained lesions was performed on sections starting just beyond the end of the aortic sinus. The lipid‐stained lesions were measured by digitizing morphometry and reported in mm^2^ per lesion.

### Cell Culture and Transfection

The human monocyte leukemia cell line THP‐1 was purchased from the Cell Bank of the Chinese Academy of Science. Foam cells were induced from the human monocyte leukemia cell line THP‐1 as described previously. Briefly, the treatment of THP‐1 cells with 100 nmol L^−1^ phorbol‐12‐myristate‐13‐acetate (PMA) (Promega, Madison, WI) for 24 h led to the differentiation of the THP‐1 cells into macrophages. The macrophages were then stimulated with 50 mg mL^−1^ ox‐LDL (Yiyuan Biotechnologies, Guangzhou, China) for 48 h to induce the transformation of macrophages into foam cells. The OE‐circ‐PIAS1‐5, si‐circ‐PIAS1‐5, recombinant adenoviruses expressing TEAD1, and YTHDC1 were purchased from HANBIO (Shanghai, China), the miR‐219a‐2‐3p mimic and inhibitor were synthesized by GenePharma (Shanghai, China), and they were transfected into foam cells according to the manufacturer's protocol.

### Circular RNA Sequencing and Annotation

Total RNA was isolated from foam cells by using TRIzol (Invitrogen), and quality and quantity were assessed via an Agilent 2100 bioanalyzer. Ribosomal RNA was removed via the rRNA Depletion Nano Kit (Qiagen), and cDNA was prepared and amplified using the Ovation RNA‐Seq System V2 (NuGEN) Kit following the manufacturer's instructions. The amplified cDNA was fragmented using a Bioruptor (Diagenode), adaptors were ligated to cDNA using TruSeq ChIP Sample Preparation Kit (Illumina, San Diego, CA, USA), the DNA fragments were size‐selected (300–350 bp) after electrophoresis on a 2.5% agarose gel, and the selected DNA was subjected to 17 cycles of PCR amplification. Library quality was determined on a Bioanalyzer 2100, and the final libraries were sequenced using the Illumina HiSeq 2500 instrument.

### Circular RNA Data Analysis

For circRNA‐Seq analysis, adapter contaminants were removed from the raw FASTQ files, and sequences were aligned to the human genome (hg19) with TopHat2 (v2.1.0), first to identify linear RNA and later to identify fusion transcripts via reads that did not align to the linear RNA. The CIRC explorer program was run with the fusion transcripts obtained from TopHat2 via Ensemble GRCh37 Release 82 annotation to identify the circular RNAs. Additionally, the cleaned FASTQ files (after adapters were removed) were used to find circular junctions via the Finding CircRNAs software. In brief, the reads were aligned to linear RNA via the Bowtie 2 program, and the unmapped reads were subsequently split into two anchors and aligned via Bowtie 2, followed by the identification of circularizing junctions. Using CIRC explorer and circRNA analysis, the combined circRNA junction read numbers from two samples were normalized to the respective number of mapped reads and represented as “reads per million.”

### Sanger Sequencing

Agarose gel electrophoresis revealed that the PCR amplification products were inserted into a T‐vector for sequencing to confirm the amplicons. The primers were synthesized, and Sanger sequencing was performed by Sangon Biotech (Shanghai, China).

### RNase R Assay and Actinomycin D Treatment

Total RNA (2 µg) was incubated with or without 3 U µg^−1^ RNase R (Epicenter, Madison, WI, USA) at 37 °C for 20 min. RT‐qPCR was then used to amplify the circRNA via specific divergent primers for the back‐splice junction of circ‐PIAS1‐5. Moreover, foam cells were treated with 2 µg mL^−1^ actinomycin D (Sigma–Aldrich, St. Louis, MO, USA) or dimethyl sulfoxide (DMSO) (Sigma–Aldrich) for 12 h. After the cells were harvested, the stability of circ‐PIAS1‐5 and linear RNA PIAS1 was tested via qRT‐qPCR.

### RNA‐FISH

A circ‐PIAS1‐5 FISH probe and a fluorescent in situ hybridization kit purchased from RiboBio (Guangzhou, China) were used for RNA‐FISH to identify the location and expression of circ‐PIAS1‐5 in foam cells and frozen sections of aortic roots. Hybridization conditions and imaging were performed as described previously. For the aortic root sections, the immunofluorescence staining protocol was performed as described above. The primer and primer sequences are listed in Table S1 (Supporting Information).

### Immunofluorescence Staining

The aortic roots were frozen in liquid nitrogen and embedded in Tissue‐Tek OCT compound (Sakura Finetek USA, Inc., Torrance, CA, USA). Frozen tissue sections (7.0 µm thick) were fixed in 4% paraformaldehyde, permeabilized in 0.2% Triton X‐100, blocked with 10% goat serum, and then incubated with the indicated primary antibodies (Abcam, Cambridge, MA) at 4 °C overnight. After being washed with 0.1% Tween‐20 phosphate buffer saline (PBS) three times, the tissue sections were incubated with fluorescein‐conjugated secondary antibodies (Abcam, Cambridge, MA) for 2 h at room temperature, followed by staining with 4,6‐diamidino‐2‐phenylindole (DAPI, Bioss) for 5 min at room temperature. The fluorescence was detected and photographed via confocal microscopy (Carl Zeiss LSM 800; Olympus, Japan).

### qRT‐PCR Analysis

Total RNA was purified from tissues or cultured cells via TRIzol reagent (Invitrogen, Carlsbad, CA, USA). For qRT‐PCR, RNA was reverse transcribed to cDNA via a reverse transcription kit (Takara, Dalian, China). Real‐time PCR analysis was performed using SYBR Green (Takara, Dalian China). The results were normalized to the expression of glyceraldehyde‐3‐phosphate dehydrogenase (GAPDH). The primers used for circ‐PIAS1‐5, YTHDC1, TEAD1, perilipin, adipophilin, and TIP47 are listed in Table S2 (Supporting Information). Bulge‐Loop miR primers for U6 and miR‐219a‐2‐3p (RIBOBIO, China) were used according to the manufacturer's protocols. U6 was used as a control for normalization. The relative quantification of the PCR products was performed according to the 2^−ΔΔ^ *^ct^* method, and the results were normalized to those of the control.

### Western Blot Analysis

The same amount of protein (30 mg) from each sample was loaded and electrophoresed via sodium dodecyl sulfate‐polyacrylamide gel electrophoresis (SDS‐PAGE) (10% polyacrylamide), transferred to nitrocellulose membranes at 300 mA for 2 h at 4 °C with gentle agitation on a platform shaker and washed three times for 5 min each in PBS with 0.1% Tween‐20 (PBST). The membrane was then incubated with a monoclonal antibody (1:1000 dilution) (Abcam, Cambridge, MA, USA) at 4 °C overnight. β‐actin was used as a loading control. The complex was subsequently washed three times with PBST and incubated with a secondary antibody (goat antirabbit horseradish peroxidase‐conjugated immunoglobulin G (1:5000 dilution) (Abcam, Cambridge, MA, USA) for 2 h at room temperature. After being washed three times with PBST, the immunoreactive protein bands were detected with an enhanced chemiluminescence (ECL) solution, and the protein bands were quantified by Image Lab (Bio‐Rad, USA).

### RNA Immunoprecipitation

The binding between circ‐PIAS1‐5 and YTHDC1 was explored by using an RNA‐Binding Protein Immunoprecipitation Kit (EMD Millipore, USA) according to the manufacturer's instructions. The cells were lysed, and the resulting lysis mixtures were subsequently incubated with antibodies against YTHDC1 or isotype control IgG. The circ‐PIAS1‐5‐YTHDC1 complexes were immunoprecipitated with protein A agarose beads, and circ‐PIAS1‐5 was extracted and purified. Subsequently, qRT‐qPCR was employed to assess the relative abundance of circ‐PIAS1‐5, with GAPDH serving as the internal control.

### RNA Pulldown

A Pierce Magnetic RNA‐Protein Pull‐Down Kit (cat. no., Thermo Scientific, 20164) was used to perform the RNA–protein pulldown assay. The biotin‐coupled RNA complex was pulled down by incubating the cell lysates with streptavidin‐coated magnetic beads (Invitrogen, Carlsbad, USA) following the manufacturer's instructions. The enrichment of circ‐PIAS1‐5 in the capture fractions was evaluated by qRT‐PCR analysis. The bound proteins were eluted from the packed beads and analyzed by SDS‐PAGE. circ‐PIAS1‐5 junction probe: 5′‐AAGUUCUUCAGGAUACCUUAUGAUGAGGCCGCACGUUGA GGA‐3′; control probe (ordered from Sangon Biotech, Shanghai, China): 5′‐UUGUACUACACAAAAGU ACUG‐3′. The RNA‐binding proteins in the capture complex were identified via western blotting, silver staining or mass spectrometry analysis.

### Luciferase Reporter Assay

The luciferase reporter constructs containing circ‐PIAS1‐5‐WT and circ‐PIAS1‐5‐Mut and wild‐type or mutant TEAD1 (TEAD1‐WT and TEAD1‐Mut) were provided by GenePharma (China). Circ‐PIAS1‐5‐WT, circ‐PIAS1‐5‐Mut, TEAD1‐WT or TEAD1‐Mut, and miR‐219a‐2‐3p mimic or miR‐NC were cotransfected into HEK293T cells via the Lipofectamine 2000 reagent (Invitrogen). Cell lysates were collected after 48 h of transfection, and luciferase reporter assays were conducted via a dual‐luciferase reporter assay system (Promega, Madison, WI, USA) according to the manufacturer's instructions. Relative luciferase activity was normalized to that of the Renilla luciferase internal control.

### Statistical Analysis

Statistical analysis was performed with GraphPad Prism 8.0 software, and images were acquired with Adobe Illustrator 2020 software. The experimental results were expressed as the mean ± standard deviation (SD). One‐way ANOVA, Student‐Newman–Kaul's test (comparisons between multiple groups), or unpaired Student's *t*‐test (between two groups) was used as appropriate. *p* < 0.05 was considered statistically significant.

## Conflict of Interest

The authors declare no conflict of interest.

## Author Contributions

S.C.M. and F.M. contributed equally to this work. S.C.M., N.L., H.P.Z., and Y.D.J. conceived and designed the research. S.C.M., F.M., N.D., YE.C., and N.L. performed the animal experiments. N.D., F.M., L.X., A.N.Y., Y.J., K.W., YE.C., J.T.X., and J.Y.S. performed all the in vitro experiments. S.C.M., F.M., N.D., and H.P.Z. performed the histological analysis. S.C.M., F.M., N.L., and Y.D.J. analyzed the data.

## Supporting information



Supporting Information

## Data Availability

All data that support the findings of this study are provided within the paper and its Supporting Information. The transcriptomes’ raw data were deposited to the NCBI Sequence Read Archive (SRA, https://www.ncbinm.nihgov/sra)with the identifierPRJNA1031148. UCSC Genome Browser was used for circ‐PIAS1‐5 (https://genome.ucsc.edu/index.html). The raw datasets generated during the study are provided within source data, which are available for all the figures and figures in the Supporting Information.
