# Supplementary material for: Homocysteine Promotes the Pathogenesis of Atherosclerosis through the Circ‐PIAS1‐5/miR‐219a‐2‐3p/TEAD1 Axis
Source: Adv Sci (Weinh). 2025 Mar 16;12(18):2415563. doi: 10.1002/advs.202415563 (PMC12079422; doi:10.1002/advs.202415563)
Supplement: Supplementary file 1 — Supporting Information [file ADVS-12-2415563-s001.docx]

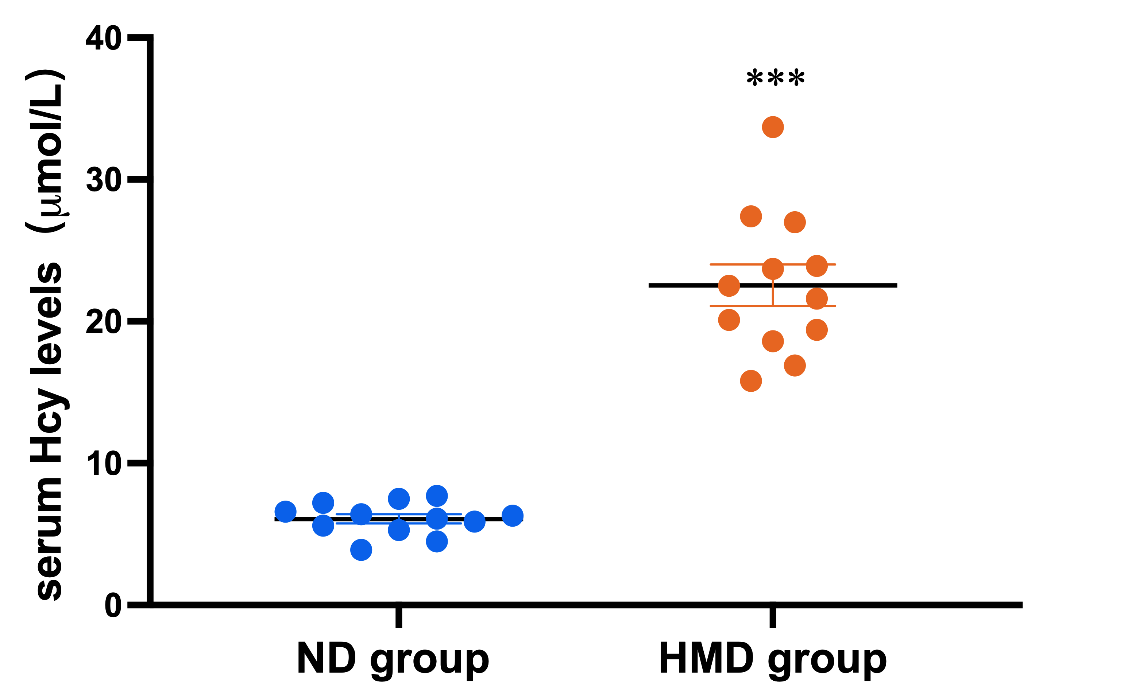


**Fig. S1 The plasma levels of Hcy in the *ApoE^−/−^* mice were measured by an automatic biochemistry analyzer**. ^***^*P<0.0001*, compared with the ND group.

*^
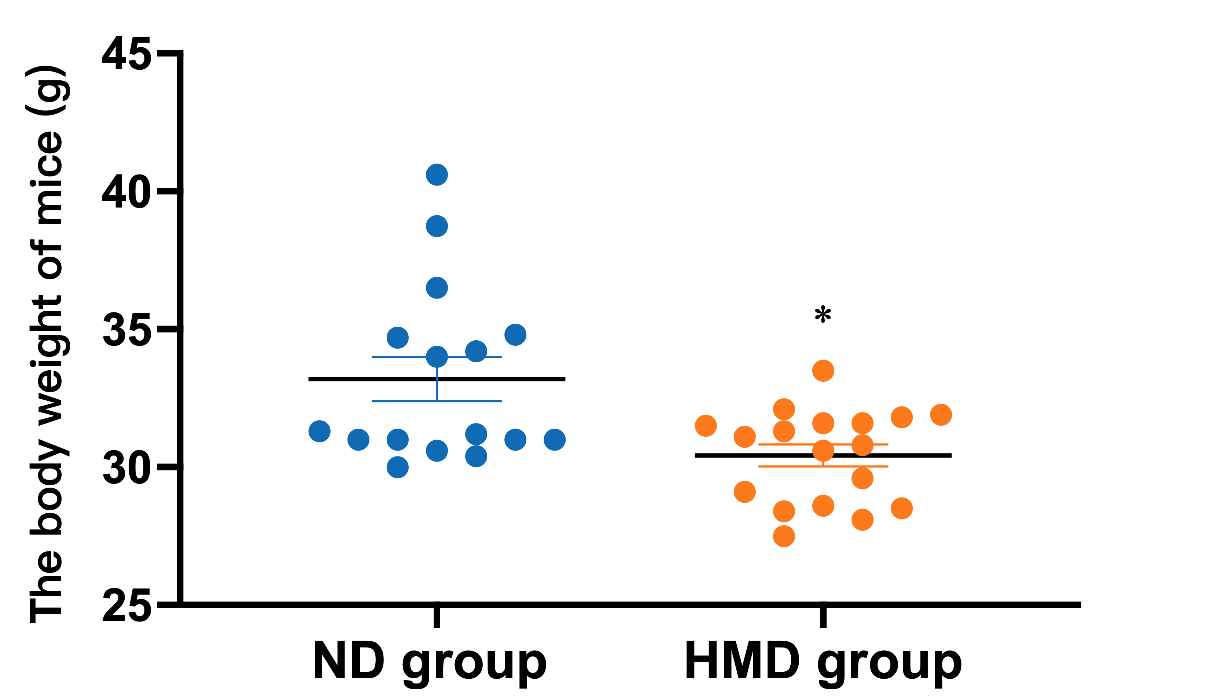
^*

**Fig. S2 The body weight of the *ApoE^−/−^* mice**. *^*^P<0.05*, compared with the ND group.


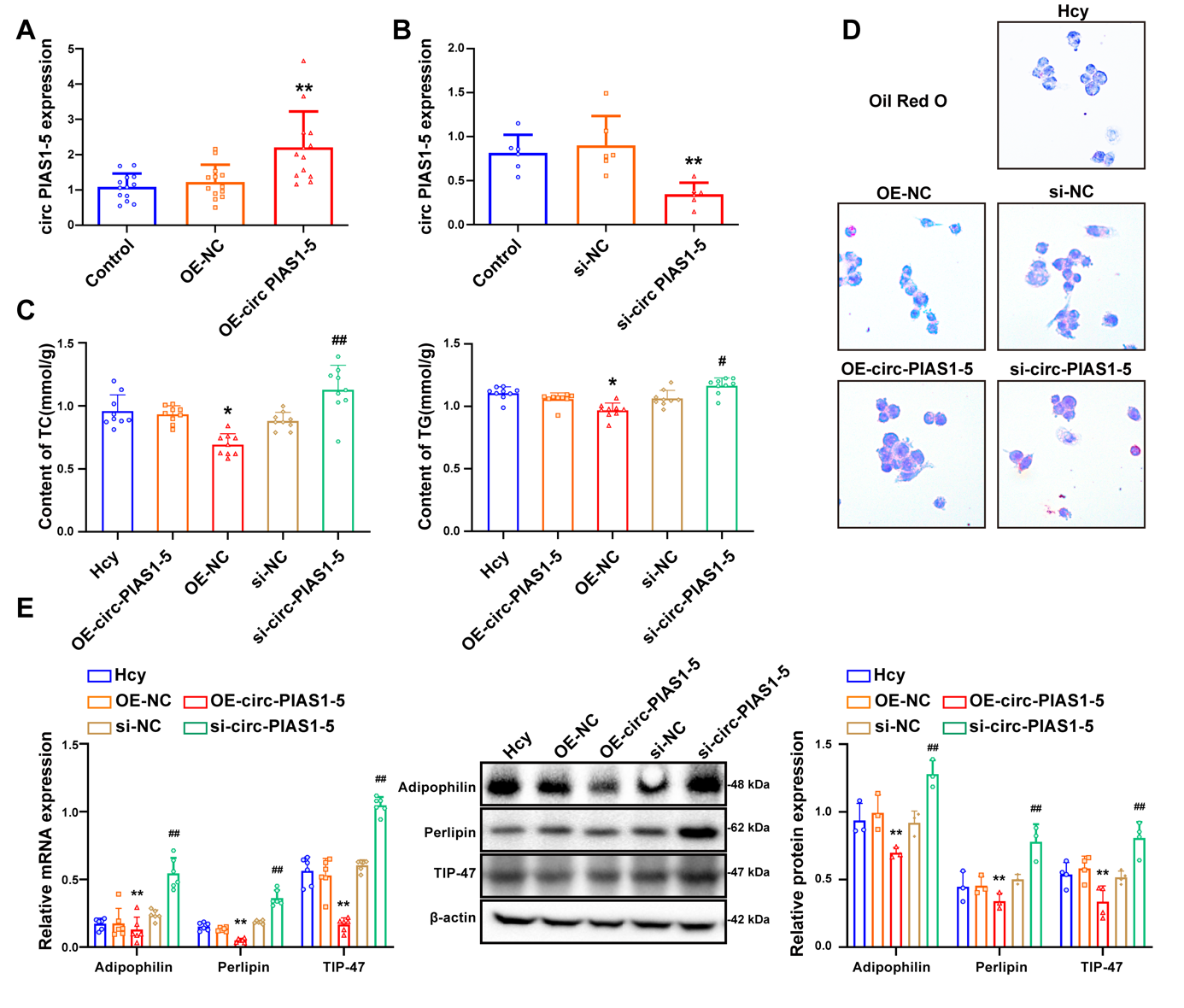


**Fig.** [**S**](javascript:;)**3 Circ-PIAS1-5 expression in foam cells. (A)** The expression of circ-PIAS1-5 was detected by qRT-PCR in foam cells transfected with circ-PIAS1-5-overexpressing vectors (OE-circ-PIAS1-5) and negative control vectors (OE-NC). **(B)** The expression of circ-PIAS1-5 in foam cells transfected with siRNAs against circ-PIAS1-5 (si-circ-PIAS1-5) and negative control siRNAs (si-NC) was measured by qRT-PCR. **(C)** TC and TG contents were detected in foam cells transfected with circ-PIAS1-5-overexpressing vectors (OE-circ-PIAS1-5) or siRNAs against circ-PIAS1-5 (si-circ-PIAS1-5) in present of Hcy. **(D)** Representative images of Oil Red O staining in foam cells transfected with OE-circ-PIAS1-5 or si-circ-PIAS1-5 in present of Hcy (scale bar=50 μm). **(E)** The expression of perilipin, adipophilin and TIP47 was determined by western blot and qRT-PCR in foam cells transfected with OE-circ-PIAS1-5 or si-circ-PIAS1-5 in present of Hcy. The data are presented as the mean±SD. ^*^*P* <0.05, ^**^*P* <0.01, compared with the Control group or Hcy group; ^##^*P*<0.01, compared with the OE-NC group or si-NC group.

**Fig.** [**S**](javascript:;)**4** The expression of circ-PIAS1-5 in the blood of healthy controls and atherosclerotic patients without HHcy was determined by qRT-PCR.


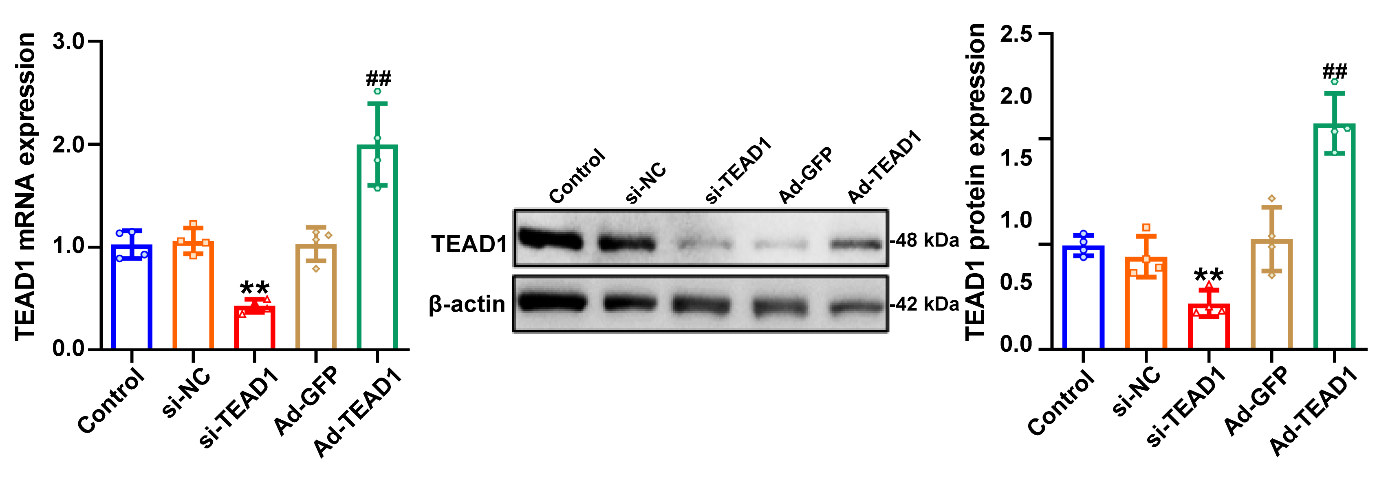


**Fig S5. The expression of TEAD1 in foam cells.** The expression of TEAD1 was detected by qRT-PCR and Western blot in foam cells transfected with TEAD1 small interfering RNA (si-TEAD1) and adenovirus overexpressing TEAD1 (Ad-TEAD1), respectively. Data were presented as Mean±SD. ^**^*P*<0.01, compared with si-NC group; ^##^*P*<0.01, compared with Ad-TEAD1 group.

**Table S1**. Primer sequences for RNA FISH

| Gene  (Genbank) | Sequences |
| --- | --- |
| circ-PIAS1-5 | Probe:5'-6-FAM-GCTTGGTAAAAAAGTGTGGG-TAMRA-3' |
| YTHDC1 | Probe:5'-6-FAM-TGGAGGACCAGCTGAAGCCCA-TAMRA-3' |
| miR-219-2-3p | Probe:5'-6-FAM-GCTTGAGGAAGAGGAAGGACA-TAMRA-3' |

**Table S2**. Primer sequences for real-time quantitative PCR (qRT-PCR) analysis

| Gene  (Genbank) | Sequences | Tm | Length  (bp) |
| --- | --- | --- | --- |
| circ-PIAS1-5  (NM_007644) | Forword:5'-CAAAAATCGTGGAGTGGA-3'  Reserve:5'-TACCGAAAAGCAGGCAGT-3' | 53.8℃ | 208 |
| YTHDC1  (NM_00100396) | Forword:5'-ACCATGCTCTGGAGAAAGCTA-3'  Reserve:5'-TGGTTGCGTGTTGTTGGGTT-3' | 59.2℃ | 197 |
| TEAD1  (NM_021961.6) | Forward:5'-ATGGAAAGGATGAGTGACTCTGC-3' | 61.7℃ | 118 |
|  | Reserve:5'-TCCCACATGGTGGATAGATAGC-3' |  |  |
| Perilipin  (NM_001145311.2) | Forward:5'-TGTGCAATGCCTATGAGAAGG-3' | 61.4℃ | 154 |
|  | Reserve:5'-AGGGCGGGGATCTTTTCCT-3' |  |  |
| Adipophilin  (NM_001122.4) | Forward:5'-ATGGCATCCGTTGCAGTTGAT-3' | 62.3℃ | 90 |
|  | Reserve:5'-GGACATGAGGTCATACGTGGAG-3' |  |  |
| TIP47  (NM_001164189.2) | Forward:5'-TATGCCTCCACCAAGGAGAG-3' | 61.4℃ | 148 |
|  | Reserve:5'-ATTCGCTGGCTGATGCAATCT-3' |  |  |
